# Supplementary material for: Repurposing Anti-Dengue Compounds against Monkeypox Virus Targeting Core Cysteine Protease
Source: Biomedicines. 2023 Jul 18;11(7):2025. doi: 10.3390/biomedicines11072025 (PMC10377189; doi:10.3390/biomedicines11072025)
Supplement: Supplementary file 1 [file biomedicines-11-02025-s001.zip › Supplementary Table S1-2.pdf]

Supplementary Table S1. The binding energies of the 1467 anti-dengue compounds against the clusters of the MPXV cysteine proteinase.

Compounds	Binding energy (kcal/mol) from docking with Cluster-1	Compounds	Binding energy (kcal/mol) from docking with Cluster-2	Compounds	Binding energy (kcal/mol) from docking with Cluster-3
drug1176	-10.5	drug562	-10.7	drug1185	-10.9
drug1023	-10.2	drug956	-10.4	drug1099	-10.8
drug1272	-10	drug1315	-10.2	drug1186	-10.8
drug1309	-10	drug935	-10.1	drug1225	-10.8
drug1084	-9.9	drug1489	-10.1	drug1273	-10.8
drug1125	-9.8	drug678	-10	drug1187	-10.7
drug367	-9.7	drug1023	-9.9	drug1272	-10.7
drug1005	-9.7	drug269	-9.8	drug1006	-10.6
drug260	-9.6	drug1084	-9.8	drug821	-10.4
drug261	-9.6	drug234	-9.7	drug995	-10.4
drug404	-9.6	drug324	-9.7	drug1270	-10.4
drug493	-9.5	drug438	-9.7	drug1441	-10.4
drug694	-9.5	drug489	-9.7	drug1471	-10.4
drug1018	-9.5	drug152	-9.6	drug1155	-10.3
drug1154	-9.5	drug410	-9.6	drug1100	-10.1
drug1186	-9.5	drug441	-9.6	drug1269	-10.1
drug1187	-9.5	drug760	-9.5	drug1271	-10.1
drug1188	-9.5	drug1308	-9.5	drug733	-10
drug1269	-9.5	drug1335	-9.5	drug994	-10
drug1273	-9.5	drug1441	-9.5	drug1018	-10
drug1441	-9.5	drug57	-9.4	drug1154	-10
drug1006	-9.4	drug421	-9.4	drug1184	-10
drug1099	-9.4	drug464	-9.4	drug1268	-10
drug1183	-9.4	drug484	-9.4	drug1309	-10
drug416	-9.3	drug505	-9.4	drug404	-9.9
drug733	-9.3	drug536	-9.4	drug1183	-9.8
drug748	-9.3	drug733	-9.4	drug42	-9.7
drug1100	-9.3	drug817	-9.4	drug622	-9.7
drug1155	-9.3	drug874	-9.4	drug1005	-9.7
drug1225	-9.3	drug1063	-9.4	drug1176	-9.7
drug1268	-9.3	drug1100	-9.4	drug1023	-9.5
drug1271	-9.3	drug1187	-9.4	drug1060	-9.5
drug1471	-9.3	drug1225	-9.4	drug1150	-9.5
drug678	-9.2	drug1268	-9.4	drug1442	-9.5
drug731	-9.2	drug1271	-9.4	drug1467	-9.5
drug1076	-9.2	drug1272	-9.4	drug261	-9.4
drug484	-9.1	drug1273	-9.4	drug611	-9.4
drug533	-9.1	drug323	-9.3	drug874	-9.4
drug562	-9.1	drug368	-9.3	drug1125	-9.4
drug730	-9.1	drug380	-9.3	drug1189	-9.4

drug817	-9.1	drug416	-9.3	drug1274	-9.4
drug874	-9.1	drug463	-9.3	drug1489	-9.4
drug1052	-9.1	drug465	-9.3	drug484	-9.3
drug1184	-9.1	drug494	-9.3	drug581	-9.3
drug1185	-9.1	drug508	-9.3	drug646	-9.3
drug1196	-9.1	drug653	-9.3	drug997	-9.3
drug1410	-9.1	drug691	-9.3	drug1069	-9.3
drug1421	-9.1	drug936	-9.3	drug1233	-9.3
drug235	-9	drug1006	-9.3	drug417	-9.2
drug443	-9	drug1028	-9.3	drug493	-9.2
drug466	-9	drug1029	-9.3	drug508	-9.2
drug508	-9	drug1069	-9.3	drug562	-9.2
drug646	-9	drug1121	-9.3	drug648	-9.2
drug695	-9	drug1185	-9.3	drug678	-9.2
drug775	-9	drug1249	-9.3	drug694	-9.2
drug1195	-9	drug1258	-9.3	drug898	-9.2
drug1258	-9	drug1468	-9.3	drug956	-9.2
drug1274	-9	drug1496	-9.3	drug1188	-9.2
drug1339	-9	drug219	-9.2	drug414	-9.1
drug24	-8.9	drug233	-9.2	drug443	-9.1
drug139	-8.9	drug347	-9.2	drug479	-9.1
drug204	-8.9	drug350	-9.2	drug482	-9.1
drug414	-8.9	drug392	-9.2	drug834	-9.1
drug618	-8.9	drug409	-9.2	drug852	-9.1
drug855	-8.9	drug429	-9.2	drug924	-9.1
drug1069	-8.9	drug440	-9.2	drug986	-9.1
drug1121	-8.9	drug443	-9.2	drug1121	-9.1
drug1270	-8.9	drug543	-9.2	drug1241	-9.1
drug126	-8.8	drug546	-9.2	drug1315	-9.1
drug199	-8.8	drug640	-9.2	drug1333	-9.1
drug289	-8.8	drug864	-9.2	drug1381	-9.1
drug421	-8.8	drug261	-9.1	drug1420	-9.1
drug430	-8.8	drug288	-9.1	drug112	-9
drug444	-8.8	drug332	-9.1	drug270	-9
drug463	-8.8	drug363	-9.1	drug625	-9
drug494	-8.8	drug411	-9.1	drug1084	-9
drug527	-8.8	drug877	-9.1	drug1195	-9
drug581	-8.8	drug882	-9.1	drug1310	-9
drug692	-8.8	drug919	-9.1	drug57	-8.9
drug704	-8.8	drug1113	-9.1	drug173	-8.9
drug716	-8.8	drug1154	-9.1	drug320	-8.9
drug935	-8.8	drug1166	-9.1	drug331	-8.9
drug1166	-8.8	drug1176	-9.1	drug391	-8.9
drug1194	-8.8	drug1186	-9.1	drug444	-8.9
drug1263	-8.8	drug1288	-9.1	drug465	-8.9
drug1307	-8.8	drug1490	-9.1	drug692	-8.9
drug1408	-8.8	drug21	-9	drug855	-8.9

drug1411	-8.8	drug42	-9	drug883	-8.9
drug1428	-8.8	drug50	-9	drug1129	-8.9
drug10	-8.7	drug95	-9	drug1232	-8.9
drug80	-8.7	drug112	-9	drug1421	-8.9
drug110	-8.7	drug170	-9	drug1428	-8.9
drug112	-8.7	drug250	-9	drug1493	-8.9
drug191	-8.7	drug342	-9	drug26	-8.8
drug340	-8.7	drug375	-9	drug44	-8.8
drug352	-8.7	drug379	-9	drug260	-8.8
drug380	-8.7	drug391	-9	drug343	-8.8
drug464	-8.7	drug493	-9	drug352	-8.8
drug465	-8.7	drug495	-9	drug446	-8.8
drug513	-8.7	drug532	-9	drug460	-8.8
drug653	-8.7	drug615	-9	drug546	-8.8
drug735	-8.7	drug624	-9	drug624	-8.8
drug776	-8.7	drug652	-9	drug730	-8.8
drug818	-8.7	drug694	-9	drug818	-8.8
drug856	-8.7	drug775	-9	drug820	-8.8
drug882	-8.7	drug867	-9	drug928	-8.8
drug904	-8.7	drug978	-9	drug935	-8.8
drug918	-8.7	drug998	-9	drug987	-8.8
drug936	-8.7	drug1093	-9	drug989	-8.8
drug1113	-8.7	drug1117	-9	drug1052	-8.8
drug1150	-8.7	drug1129	-9	drug1182	-8.8
drug1189	-8.7	drug1269	-9	drug1328	-8.8
drug1233	-8.7	drug1307	-9	drug1410	-8.8
drug1315	-8.7	drug1428	-9	drug1435	-8.8
drug1381	-8.7	drug1454	-9	drug1444	-8.8
drug1420	-8.7	drug193	-8.9	drug1490	-8.8
drug1427	-8.7	drug270	-8.9	drug10	-8.7
drug1489	-8.7	drug275	-8.9	drug24	-8.7
drug324	-8.6	drug320	-8.9	drug169	-8.7
drug345	-8.6	drug339	-8.9	drug193	-8.7
drug350	-8.6	drug344	-8.9	drug235	-8.7
drug351	-8.6	drug362	-8.9	drug403	-8.7
drug392	-8.6	drug462	-8.9	drug428	-8.7
drug417	-8.6	drug496	-8.9	drug450	-8.7
drug476	-8.6	drug512	-8.9	drug466	-8.7
drug523	-8.6	drug679	-8.9	drug485	-8.7
drug543	-8.6	drug762	-8.9	drug512	-8.7
drug620	-8.6	drug948	-8.9	drug533	-8.7
drug734	-8.6	drug989	-8.9	drug578	-8.7
drug766	-8.6	drug1060	-8.9	drug716	-8.7
drug770	-8.6	drug1073	-8.9	drug723	-8.7
drug851	-8.6	drug1079	-8.9	drug736	-8.7
drug873	-8.6	drug1099	-8.9	drug830	-8.7
drug883	-8.6	drug1155	-8.9	drug864	-8.7

drug986	-8.6	drug1156	-8.9	drug961	-8.7
drug994	-8.6	drug1184	-8.9	drug977	-8.7
drug997	-8.6	drug1330	-8.9	drug988	-8.7
drug1053	-8.6	drug1460	-8.9	drug1007	-8.7
drug1079	-8.6	drug1488	-8.9	drug1043	-8.7
drug1095	-8.6	drug55	-8.8	drug1079	-8.7
drug1208	-8.6	drug68	-8.8	drug1204	-8.7
drug1351	-8.6	drug81	-8.8	drug1216	-8.7
drug1370	-8.6	drug118	-8.8	drug1370	-8.7
drug1406	-8.6	drug301	-8.8	drug1371	-8.7
drug1415	-8.6	drug309	-8.8	drug1425	-8.7
drug1442	-8.6	drug334	-8.8	drug1468	-8.7
drug1467	-8.6	drug369	-8.8	drug1474	-8.7
drug42	-8.5	drug398	-8.8	drug1	-8.6
drug57	-8.5	drug430	-8.8	drug33	-8.6
drug173	-8.5	drug444	-8.8	drug103	-8.6
drug188	-8.5	drug498	-8.8	drug110	-8.6
drug219	-8.5	drug499	-8.8	drug118	-8.6
drug343	-8.5	drug507	-8.8	drug152	-8.6
drug429	-8.5	drug593	-8.8	drug209	-8.6
drug441	-8.5	drug661	-8.8	drug220	-8.6
drug511	-8.5	drug744	-8.8	drug324	-8.6
drug546	-8.5	drug757	-8.8	drug340	-8.6
drug625	-8.5	drug924	-8.8	drug345	-8.6
drug638	-8.5	drug1188	-8.8	drug350	-8.6
drug741	-8.5	drug1202	-8.8	drug392	-8.6
drug749	-8.5	drug1240	-8.8	drug393	-8.6
drug760	-8.5	drug1242	-8.8	drug410	-8.6
drug852	-8.5	drug1279	-8.8	drug429	-8.6
drug928	-8.5	drug1343	-8.8	drug437	-8.6
drug937	-8.5	drug1347	-8.8	drug463	-8.6
drug1182	-8.5	drug1372	-8.8	drug464	-8.6
drug1193	-8.5	drug1420	-8.8	drug469	-8.6
drug1240	-8.5	drug1434	-8.8	drug489	-8.6
drug1294	-8.5	drug1467	-8.8	drug494	-8.6
drug1328	-8.5	drug1471	-8.8	drug506	-8.6
drug1343	-8.5	drug1491	-8.8	drug510	-8.6
drug1422	-8.5	drug44	-8.7	drug517	-8.6
drug1435	-8.5	drug52	-8.7	drug618	-8.6
drug1456	-8.5	drug79	-8.7	drug632	-8.6
drug1460	-8.5	drug109	-8.7	drug651	-8.6
drug1468	-8.5	drug171	-8.7	drug770	-8.6
drug26	-8.4	drug185	-8.7	drug776	-8.6
drug79	-8.4	drug385	-8.7	drug937	-8.6
drug135	-8.4	drug476	-8.7	drug1051	-8.6
drug234	-8.4	drug486	-8.7	drug1110	-8.6
drug347	-8.4	drug513	-8.7	drug1166	-8.6

drug379	-8.4	drug520	-8.7	drug1208	-8.6
drug381	-8.4	drug578	-8.7	drug1248	-8.6
drug410	-8.4	drug584	-8.7	drug1280	-8.6
drug449	-8.4	drug600	-8.7	drug1340	-8.6
drug460	-8.4	drug605	-8.7	drug1408	-8.6
drug461	-8.4	drug608	-8.7	drug1411	-8.6
drug502	-8.4	drug609	-8.7	drug1431	-8.6
drug512	-8.4	drug641	-8.7	drug121	-8.5
drug600	-8.4	drug692	-8.7	drug127	-8.5
drug611	-8.4	drug721	-8.7	drug138	-8.5
drug621	-8.4	drug793	-8.7	drug191	-8.5
drug652	-8.4	drug818	-8.7	drug212	-8.5
drug714	-8.4	drug847	-8.7	drug347	-8.5
drug754	-8.4	drug894	-8.7	drug353	-8.5
drug771	-8.4	drug906	-8.7	drug367	-8.5
drug820	-8.4	drug937	-8.7	drug379	-8.5
drug892	-8.4	drug1076	-8.7	drug416	-8.5
drug898	-8.4	drug1110	-8.7	drug421	-8.5
drug977	-8.4	drug1144	-8.7	drug430	-8.5
drug1092	-8.4	drug1160	-8.7	drug447	-8.5
drug1098	-8.4	drug1183	-8.7	drug487	-8.5
drug1110	-8.4	drug1241	-8.7	drug513	-8.5
drug1136	-8.4	drug1256	-8.7	drug527	-8.5
drug1207	-8.4	drug1263	-8.7	drug557	-8.5
drug1310	-8.4	drug1270	-8.7	drug626	-8.5
drug1313	-8.4	drug1289	-8.7	drug627	-8.5
drug1340	-8.4	drug1295	-8.7	drug639	-8.5
drug1379	-8.4	drug1325	-8.7	drug668	-8.5
drug1448	-8.4	drug1328	-8.7	drug695	-8.5
drug1454	-8.4	drug1331	-8.7	drug703	-8.5
drug1466	-8.4	drug22	-8.6	drug720	-8.5
drug1478	-8.4	drug77	-8.6	drug734	-8.5
drug1486	-8.4	drug114	-8.6	drug740	-8.5
drug1493	-8.4	drug121	-8.6	drug749	-8.5
drug53	-8.3	drug166	-8.6	drug754	-8.5
drug117	-8.3	drug173	-8.6	drug760	-8.5
drug134	-8.3	drug174	-8.6	drug878	-8.5
drug152	-8.3	drug207	-8.6	drug882	-8.5
drug193	-8.3	drug212	-8.6	drug891	-8.5
drug209	-8.3	drug220	-8.6	drug904	-8.5
drug266	-8.3	drug276	-8.6	drug978	-8.5
drug320	-8.3	drug327	-8.6	drug1097	-8.5
drug323	-8.3	drug340	-8.6	drug1145	-8.5
drug339	-8.3	drug420	-8.6	drug1160	-8.5
drug365	-8.3	drug437	-8.6	drug1258	-8.5
drug400	-8.3	drug563	-8.6	drug1263	-8.5
drug403	-8.3	drug583	-8.6	drug1307	-8.5

drug415	-8.3	drug592	-8.6	drug1308	-8.5
drug474	-8.3	drug606	-8.6	drug1313	-8.5
drug496	-8.3	drug616	-8.6	drug1335	-8.5
drug501	-8.3	drug627	-8.6	drug1399	-8.5
drug624	-8.3	drug662	-8.6	drug1412	-8.5
drug626	-8.3	drug687	-8.6	drug1460	-8.5
drug627	-8.3	drug730	-8.6	drug1496	-8.5
drug648	-8.3	drug771	-8.6	drug155	-8.4
drug674	-8.3	drug799	-8.6	drug185	-8.4
drug679	-8.3	drug852	-8.6	drug243	-8.4
drug709	-8.3	drug856	-8.6	drug266	-8.4
drug713	-8.3	drug987	-8.6	drug339	-8.4
drug779	-8.3	drug1061	-8.6	drug409	-8.4
drug837	-8.3	drug1062	-8.6	drug415	-8.4
drug1007	-8.3	drug1141	-8.6	drug440	-8.4
drug1029	-8.3	drug1204	-8.6	drug448	-8.4
drug1038	-8.3	drug1207	-8.6	drug505	-8.4
drug1077	-8.3	drug1230	-8.6	drug523	-8.4
drug1116	-8.3	drug1233	-8.6	drug543	-8.4
drug1168	-8.3	drug1239	-8.6	drug580	-8.4
drug1222	-8.3	drug1262	-8.6	drug614	-8.4
drug1232	-8.3	drug1287	-8.6	drug638	-8.4
drug1308	-8.3	drug1340	-8.6	drug660	-8.4
drug1323	-8.3	drug1356	-8.6	drug709	-8.4
drug1336	-8.3	drug1404	-8.6	drug732	-8.4
drug1357	-8.3	drug1435	-8.6	drug777	-8.4
drug1394	-8.3	drug58	-8.5	drug873	-8.4
drug1412	-8.3	drug59	-8.5	drug936	-8.4
drug1497	-8.3	drug117	-8.5	drug971	-8.4
drug21	-8.2	drug172	-8.5	drug1092	-8.4
drug27	-8.2	drug188	-8.5	drug1098	-8.4
drug52	-8.2	drug189	-8.5	drug1101	-8.4
drug166	-8.2	drug210	-8.5	drug1102	-8.4
drug267	-8.2	drug230	-8.5	drug1111	-8.4
drug309	-8.2	drug235	-8.5	drug1113	-8.4
drug313	-8.2	drug266	-8.5	drug1194	-8.4
drug353	-8.2	drug289	-8.5	drug1218	-8.4
drug369	-8.2	drug313	-8.5	drug1261	-8.4
drug388	-8.2	drug333	-8.5	drug1321	-8.4
drug409	-8.2	drug345	-8.5	drug1379	-8.4
drug411	-8.2	drug352	-8.5	drug1430	-8.4
drug447	-8.2	drug407	-8.5	drug1500	-8.4
drug482	-8.2	drug458	-8.5	drug48	-8.3
drug485	-8.2	drug511	-8.5	drug50	-8.3
drug487	-8.2	drug533	-8.5	drug147	-8.3
drug506	-8.2	drug588	-8.5	drug164	-8.3
drug507	-8.2	drug622	-8.5	drug171	-8.3

drug520	-8.2	drug625	-8.5	drug188	-8.3
drug556	-8.2	drug710	-8.5	drug192	-8.3
drug578	-8.2	drug749	-8.5	drug201	-8.3
drug632	-8.2	drug754	-8.5	drug204	-8.3
drug676	-8.2	drug768	-8.5	drug219	-8.3
drug691	-8.2	drug830	-8.5	drug234	-8.3
drug821	-8.2	drug844	-8.5	drug380	-8.3
drug829	-8.2	drug846	-8.5	drug407	-8.3
drug907	-8.2	drug866	-8.5	drug411	-8.3
drug909	-8.2	drug868	-8.5	drug456	-8.3
drug948	-8.2	drug870	-8.5	drug486	-8.3
drug989	-8.2	drug881	-8.5	drug488	-8.3
drug1012	-8.2	drug910	-8.5	drug584	-8.3
drug1074	-8.2	drug971	-8.5	drug620	-8.3
drug1082	-8.2	drug974	-8.5	drug640	-8.3
drug1097	-8.2	drug976	-8.5	drug653	-8.3
drug1114	-8.2	drug977	-8.5	drug679	-8.3
drug1153	-8.2	drug1039	-8.5	drug704	-8.3
drug1249	-8.2	drug1168	-8.5	drug731	-8.3
drug1266	-8.2	drug1216	-8.5	drug738	-8.3
drug1333	-8.2	drug1253	-8.5	drug779	-8.3
drug1359	-8.2	drug1275	-8.5	drug814	-8.3
drug1367	-8.2	drug1276	-8.5	drug906	-8.3
drug1384	-8.2	drug1283	-8.5	drug910	-8.3
drug1413	-8.2	drug1291	-8.5	drug926	-8.3
drug1437	-8.2	drug1348	-8.5	drug1029	-8.3
drug1439	-8.2	drug1	-8.4	drug1063	-8.3
drug1496	-8.2	drug54	-8.4	drug1168	-8.3
drug1	-8.1	drug69	-8.4	drug1178	-8.3
drug54	-8.1	drug126	-8.4	drug1196	-8.3
drug68	-8.1	drug164	-8.4	drug1215	-8.3
drug127	-8.1	drug209	-8.4	drug1230	-8.3
drug170	-8.1	drug282	-8.4	drug1288	-8.3
drug192	-8.1	drug353	-8.4	drug1294	-8.3
drug201	-8.1	drug376	-8.4	drug1345	-8.3
drug233	-8.1	drug381	-8.4	drug1357	-8.3
drug269	-8.1	drug386	-8.4	drug1469	-8.3
drug333	-8.1	drug404	-8.4	drug1475	-8.3
drug366	-8.1	drug405	-8.4	drug80	-8.2
drug391	-8.1	drug497	-8.4	drug81	-8.2
drug428	-8.1	drug523	-8.4	drug172	-8.2
drug446	-8.1	drug551	-8.4	drug313	-8.2
drug448	-8.1	drug585	-8.4	drug319	-8.2
drug450	-8.1	drug586	-8.4	drug323	-8.2
drug451	-8.1	drug618	-8.4	drug351	-8.2
drug579	-8.1	drug643	-8.4	drug373	-8.2
drug582	-8.1	drug703	-8.4	drug381	-8.2

drug586	-8.1	drug704	-8.4	drug385	-8.2
drug619	-8.1	drug714	-8.4	drug386	-8.2
drug634	-8.1	drug726	-8.4	drug438	-8.2
drug639	-8.1	drug732	-8.4	drug441	-8.2
drug651	-8.1	drug770	-8.4	drug451	-8.2
drug675	-8.1	drug779	-8.4	drug497	-8.2
drug705	-8.1	drug829	-8.4	drug500	-8.2
drug721	-8.1	drug848	-8.4	drug501	-8.2
drug723	-8.1	drug912	-8.4	drug502	-8.2
drug736	-8.1	drug920	-8.4	drug520	-8.2
drug767	-8.1	drug939	-8.4	drug551	-8.2
drug774	-8.1	drug940	-8.4	drug600	-8.2
drug836	-8.1	drug945	-8.4	drug608	-8.2
drug871	-8.1	drug965	-8.4	drug615	-8.2
drug877	-8.1	drug972	-8.4	drug617	-8.2
drug905	-8.1	drug973	-8.4	drug631	-8.2
drug939	-8.1	drug980	-8.4	drug656	-8.2
drug963	-8.1	drug995	-8.4	drug669	-8.2
drug978	-8.1	drug1007	-8.4	drug746	-8.2
drug998	-8.1	drug1020	-8.4	drug747	-8.2
drug1043	-8.1	drug1030	-8.4	drug757	-8.2
drug1063	-8.1	drug1033	-8.4	drug799	-8.2
drug1133	-8.1	drug1098	-8.4	drug829	-8.2
drug1146	-8.1	drug1114	-8.4	drug846	-8.2
drug1147	-8.1	drug1115	-8.4	drug894	-8.2
drug1171	-8.1	drug1126	-8.4	drug939	-8.2
drug1178	-8.1	drug1127	-8.4	drug1076	-8.2
drug1282	-8.1	drug1178	-8.4	drug1082	-8.2
drug1288	-8.1	drug1215	-8.4	drug1095	-8.2
drug1387	-8.1	drug1257	-8.4	drug1115	-8.2
drug1400	-8.1	drug1320	-8.4	drug1116	-8.2
drug1416	-8.1	drug1332	-8.4	drug1132	-8.2
drug1430	-8.1	drug1370	-8.4	drug1144	-8.2
drug1431	-8.1	drug1381	-8.4	drug1169	-8.2
drug1434	-8.1	drug1386	-8.4	drug1179	-8.2
drug44	-8	drug1399	-8.4	drug1250	-8.2
drug48	-8	drug1436	-8.4	drug1266	-8.2
drug115	-8	drug1477	-8.4	drug1330	-8.2
drug124	-8	drug48	-8.3	drug1343	-8.2
drug136	-8	drug61	-8.3	drug1344	-8.2
drug147	-8	drug84	-8.3	drug1346	-8.2
drug164	-8	drug85	-8.3	drug1372	-8.2
drug189	-8	drug94	-8.3	drug1376	-8.2
drug207	-8	drug97	-8.3	drug1384	-8.2
drug220	-8	drug110	-8.3	drug1422	-8.2
drug270	-8	drug134	-8.3	drug1458	-8.2
drug291	-8	drug245	-8.3	drug1487	-8.2

drug342	-8	drug251	-8.3	drug27	-8.1
drug385	-8	drug257	-8.3	drug52	-8.1
drug393	-8	drug326	-8.3	drug54	-8.1
drug408	-8	drug351	-8.3	drug61	-8.1
drug438	-8	drug383	-8.3	drug78	-8.1
drug440	-8	drug387	-8.3	drug134	-8.1
drug479	-8	drug400	-8.3	drug269	-8.1
drug488	-8	drug448	-8.3	drug332	-8.1
drug505	-8	drug456	-8.3	drug341	-8.1
drug510	-8	drug466	-8.3	drug366	-8.1
drug522	-8	drug478	-8.3	drug384	-8.1
drug569	-8	drug502	-8.3	drug394	-8.1
drug571	-8	drug518	-8.3	drug400	-8.1
drug572	-8	drug537	-8.3	drug445	-8.1
drug622	-8	drug557	-8.3	drug449	-8.1
drug688	-8	drug565	-8.3	drug461	-8.1
drug703	-8	drug599	-8.3	drug478	-8.1
drug722	-8	drug604	-8.3	drug480	-8.1
drug738	-8	drug611	-8.3	drug481	-8.1
drug740	-8	drug634	-8.3	drug519	-8.1
drug769	-8	drug646	-8.3	drug536	-8.1
drug777	-8	drug655	-8.3	drug545	-8.1
drug794	-8	drug723	-8.3	drug623	-8.1
drug830	-8	drug796	-8.3	drug629	-8.1
drug833	-8	drug819	-8.3	drug643	-8.1
drug861	-8	drug851	-8.3	drug676	-8.1
drug864	-8	drug901	-8.3	drug705	-8.1
drug875	-8	drug918	-8.3	drug717	-8.1
drug917	-8	drug963	-8.3	drug745	-8.1
drug947	-8	drug964	-8.3	drug774	-8.1
drug956	-8	drug975	-8.3	drug817	-8.1
drug972	-8	drug981	-8.3	drug832	-8.1
drug988	-8	drug986	-8.3	drug847	-8.1
drug990	-8	drug1027	-8.3	drug848	-8.1
drug1039	-8	drug1031	-8.3	drug875	-8.1
drug1051	-8	drug1040	-8.3	drug877	-8.1
drug1078	-8	drug1077	-8.3	drug885	-8.1
drug1105	-8	drug1139	-8.3	drug963	-8.1
drug1131	-8	drug1174	-8.3	drug981	-8.1
drug1140	-8	drug1220	-8.3	drug998	-8.1
drug1156	-8	drug1232	-8.3	drug1004	-8.1
drug1202	-8	drug1248	-8.3	drug1016	-8.1
drug1209	-8	drug1339	-8.3	drug1093	-8.1
drug1221	-8	drug1355	-8.3	drug1105	-8.1
drug1230	-8	drug1367	-8.3	drug1114	-8.1
drug1241	-8	drug1402	-8.3	drug1171	-8.1
drug1257	-8	drug1408	-8.3	drug1193	-8.1

drug1279	-8	drug1427	-8.3	drug1198	-8.1
drug1280	-8	drug1442	-8.3	drug1242	-8.1
drug1304	-8	drug1470	-8.3	drug1320	-8.1
drug1321	-8	drug1473	-8.3	drug1325	-8.1
drug1335	-8	drug1478	-8.3	drug1350	-8.1
drug1344	-8	drug1500	-8.3	drug1387	-8.1
drug1358	-8	drug23	-8.2	drug1394	-8.1
drug1371	-8	drug82	-8.2	drug1402	-8.1
drug1380	-8	drug93	-8.2	drug1406	-8.1
drug1399	-8	drug139	-8.2	drug1433	-8.1
drug1414	-8	drug147	-8.2	drug1456	-8.1
drug1465	-8	drug182	-8.2	drug1491	-8.1
drug1487	-8	drug184	-8.2	drug21	-8
drug1490	-8	drug208	-8.2	drug69	-8
drug12	-7.9	drug231	-8.2	drug125	-8
drug47	-7.9	drug279	-8.2	drug233	-8
drug50	-7.9	drug325	-8.2	drug267	-8
drug59	-7.9	drug335	-8.2	drug281	-8
drug103	-7.9	drug346	-8.2	drug289	-8
drug125	-7.9	drug418	-8.2	drug315	-8
drug138	-7.9	drug447	-8.2	drug342	-8
drug155	-7.9	drug450	-8.2	drug388	-8
drug171	-7.9	drug480	-8.2	drug395	-8
drug172	-7.9	drug481	-8.2	drug406	-8
drug185	-7.9	drug501	-8.2	drug453	-8
drug216	-7.9	drug503	-8.2	drug511	-8
drug236	-7.9	drug581	-8.2	drug544	-8
drug303	-7.9	drug607	-8.2	drug588	-8
drug307	-7.9	drug669	-8.2	drug616	-8
drug341	-7.9	drug674	-8.2	drug702	-8
drug371	-7.9	drug676	-8.2	drug729	-8
drug394	-7.9	drug695	-8.2	drug750	-8
drug399	-7.9	drug701	-8.2	drug766	-8
drug402	-7.9	drug702	-8.2	drug771	-8
drug445	-7.9	drug734	-8.2	drug844	-8
drug456	-7.9	drug741	-8.2	drug856	-8
drug471	-7.9	drug774	-8.2	drug918	-8
drug478	-7.9	drug801	-8.2	drug929	-8
drug495	-7.9	drug871	-8.2	drug940	-8
drug504	-7.9	drug905	-8.2	drug948	-8
drug547	-7.9	drug921	-8.2	drug1028	-8
drug567	-7.9	drug926	-8.2	drug1053	-8
drug568	-7.9	drug927	-8.2	drug1073	-8
drug577	-7.9	drug990	-8.2	drug1127	-8
drug584	-7.9	drug997	-8.2	drug1141	-8
drug599	-7.9	drug1005	-8.2	drug1174	-8
drug623	-7.9	drug1012	-8.2	drug1180	-8

drug628	-7.9	drug1065	-8.2	drug1211	-8
drug633	-7.9	drug1101	-8.2	drug1234	-8
drug747	-7.9	drug1125	-8.2	drug1249	-8
drug750	-7.9	drug1151	-8.2	drug1304	-8
drug757	-7.9	drug1180	-8.2	drug1311	-8
drug762	-7.9	drug1255	-8.2	drug1323	-8
drug765	-7.9	drug1281	-8.2	drug1324	-8
drug819	-7.9	drug1321	-8.2	drug1339	-8
drug832	-7.9	drug1357	-8.2	drug1367	-8
drug844	-7.9	drug1368	-8.2	drug1380	-8
drug846	-7.9	drug1371	-8.2	drug1424	-8
drug847	-7.9	drug1373	-8.2	drug1434	-8
drug878	-7.9	drug1394	-8.2	drug60	-7.9
drug908	-7.9	drug1430	-8.2	drug72	-7.9
drug912	-7.9	drug1493	-8.2	drug82	-7.9
drug971	-7.9	drug24	-8.1	drug83	-7.9
drug995	-7.9	drug26	-8.1	drug139	-7.9
drug1020	-7.9	drug56	-8.1	drug174	-7.9
drug1026	-7.9	drug73	-8.1	drug183	-7.9
drug1028	-7.9	drug83	-8.1	drug197	-7.9
drug1032	-7.9	drug119	-8.1	drug207	-7.9
drug1065	-7.9	drug140	-8.1	drug210	-7.9
drug1134	-7.9	drug155	-8.1	drug236	-7.9
drug1190	-7.9	drug214	-8.1	drug272	-7.9
drug1198	-7.9	drug243	-8.1	drug275	-7.9
drug1227	-7.9	drug260	-8.1	drug282	-7.9
drug1236	-7.9	drug370	-8.1	drug309	-7.9
drug1244	-7.9	drug384	-8.1	drug359	-7.9
drug1285	-7.9	drug393	-8.1	drug402	-7.9
drug1317	-7.9	drug415	-8.1	drug405	-7.9
drug1327	-7.9	drug422	-8.1	drug471	-7.9
drug1365	-7.9	drug482	-8.1	drug507	-7.9
drug1376	-7.9	drug517	-8.1	drug532	-7.9
drug1401	-7.9	drug521	-8.1	drug540	-7.9
drug1424	-7.9	drug556	-8.1	drug605	-7.9
drug1429	-7.9	drug626	-8.1	drug641	-7.9
drug1447	-7.9	drug632	-8.1	drug701	-7.9
drug1500	-7.9	drug638	-8.1	drug710	-7.9
drug55	-7.8	drug654	-8.1	drug714	-7.9
drug58	-7.8	drug666	-8.1	drug744	-7.9
drug61	-7.8	drug696	-8.1	drug762	-7.9
drug69	-7.8	drug706	-8.1	drug765	-7.9
drug72	-7.8	drug716	-8.1	drug775	-7.9
drug81	-7.8	drug738	-8.1	drug793	-7.9
drug82	-7.8	drug758	-8.1	drug794	-7.9
drug93	-7.8	drug766	-8.1	drug866	-7.9
drug174	-7.8	drug767	-8.1	drug876	-7.9

drug184	-7.8	drug769	-8.1	drug919	-7.9
drug245	-7.8	drug795	-8.1	drug931	-7.9
drug250	-7.8	drug808	-8.1	drug964	-7.9
drug259	-7.8	drug853	-8.1	drug996	-7.9
drug282	-7.8	drug887	-8.1	drug1010	-7.9
drug335	-7.8	drug896	-8.1	drug1033	-7.9
drug355	-7.8	drug898	-8.1	drug1042	-7.9
drug386	-7.8	drug947	-8.1	drug1057	-7.9
drug419	-7.8	drug961	-8.1	drug1064	-7.9
drug453	-7.8	drug970	-8.1	drug1122	-7.9
drug481	-7.8	drug1038	-8.1	drug1136	-7.9
drug489	-7.8	drug1046	-8.1	drug1153	-7.9
drug534	-7.8	drug1052	-8.1	drug1199	-7.9
drug557	-7.8	drug1102	-8.1	drug1220	-7.9
drug593	-7.8	drug1120	-8.1	drug1244	-7.9
drug603	-7.8	drug1122	-8.1	drug1252	-7.9
drug605	-7.8	drug1194	-8.1	drug1264	-7.9
drug607	-7.8	drug1206	-8.1	drug1279	-7.9
drug614	-7.8	drug1214	-8.1	drug1347	-7.9
drug615	-7.8	drug1218	-8.1	drug1351	-7.9
drug617	-7.8	drug1236	-8.1	drug1354	-7.9
drug715	-7.8	drug1237	-8.1	drug1355	-7.9
drug720	-7.8	drug1251	-8.1	drug1356	-7.9
drug744	-7.8	drug1267	-8.1	drug1368	-7.9
drug746	-7.8	drug1274	-8.1	drug1397	-7.9
drug822	-7.8	drug1294	-8.1	drug1436	-7.9
drug876	-7.8	drug1303	-8.1	drug1439	-7.9
drug886	-7.8	drug1324	-8.1	drug1459	-7.9
drug890	-7.8	drug1337	-8.1	drug20	-7.8
drug891	-7.8	drug1365	-8.1	drug62	-7.8
drug894	-7.8	drug1385	-8.1	drug68	-7.8
drug901	-7.8	drug1421	-8.1	drug73	-7.8
drug921	-7.8	drug1425	-8.1	drug75	-7.8
drug926	-7.8	drug1439	-8.1	drug109	-7.8
drug942	-7.8	drug1465	-8.1	drug124	-7.8
drug973	-7.8	drug28	-8	drug126	-7.8
drug1002	-7.8	drug74	-8	drug140	-7.8
drug1057	-7.8	drug165	-8	drug166	-7.8
drug1060	-7.8	drug169	-8	drug182	-7.8
drug1067	-7.8	drug197	-8	drug184	-7.8
drug1073	-7.8	drug206	-8	drug250	-7.8
drug1093	-7.8	drug248	-8	drug291	-7.8
drug1101	-7.8	drug267	-8	drug327	-7.8
drug1111	-7.8	drug268	-8	drug344	-7.8
drug1169	-7.8	drug291	-8	drug361	-7.8
drug1174	-7.8	drug373	-8	drug368	-7.8
drug1180	-7.8	drug388	-8	drug369	-7.8

drug1204	-7.8	drug414	-8	drug383	-7.8
drug1212	-7.8	drug442	-8	drug408	-7.8
drug1218	-7.8	drug460	-8	drug419	-7.8
drug1231	-7.8	drug470	-8	drug458	-7.8
drug1248	-7.8	drug474	-8	drug470	-7.8
drug1281	-7.8	drug509	-8	drug496	-7.8
drug1286	-7.8	drug572	-8	drug534	-7.8
drug1287	-7.8	drug637	-8	drug537	-7.8
drug1356	-7.8	drug651	-8	drug563	-7.8
drug1368	-7.8	drug658	-8	drug585	-7.8
drug1402	-7.8	drug677	-8	drug593	-7.8
drug1485	-7.8	drug711	-8	drug606	-7.8
drug20	-7.7	drug729	-8	drug628	-7.8
drug62	-7.7	drug753	-8	drug630	-7.8
drug78	-7.7	drug832	-8	drug662	-7.8
drug118	-7.7	drug873	-8	drug735	-7.8
drug119	-7.7	drug886	-8	drug751	-7.8
drug169	-7.7	drug968	-8	drug753	-7.8
drug182	-7.7	drug985	-8	drug758	-7.8
drug187	-7.7	drug994	-8	drug767	-7.8
drug197	-7.7	drug1018	-8	drug796	-7.8
drug200	-7.7	drug1025	-8	drug884	-7.8
drug238	-7.7	drug1036	-8	drug892	-7.8
drug247	-7.7	drug1043	-8	drug905	-7.8
drug264	-7.7	drug1053	-8	drug917	-7.8
drug268	-7.7	drug1082	-8	drug980	-7.8
drug302	-7.7	drug1097	-8	drug1026	-7.8
drug326	-7.7	drug1106	-8	drug1066	-7.8
drug331	-7.7	drug1111	-8	drug1067	-7.8
drug332	-7.7	drug1119	-8	drug1117	-7.8
drug356	-7.7	drug1171	-8	drug1119	-7.8
drug378	-7.7	drug1182	-8	drug1134	-7.8
drug387	-7.7	drug1219	-8	drug1143	-7.8
drug405	-7.7	drug1277	-8	drug1200	-7.8
drug406	-7.7	drug1323	-8	drug1202	-7.8
drug437	-7.7	drug1349	-8	drug1222	-7.8
drug480	-7.7	drug1351	-8	drug1236	-7.8
drug486	-7.7	drug1383	-8	drug1240	-7.8
drug497	-7.7	drug1403	-8	drug1267	-7.8
drug532	-7.7	drug1456	-8	drug1282	-7.8
drug536	-7.7	drug1492	-8	drug1287	-7.8
drug549	-7.7	drug10	-7.9	drug1289	-7.8
drug558	-7.7	drug115	-7.9	drug1348	-7.8
drug570	-7.7	drug124	-7.9	drug1359	-7.8
drug580	-7.7	drug127	-7.9	drug1448	-7.8
drug606	-7.7	drug191	-7.9	drug1486	-7.8
drug608	-7.7	drug204	-7.9	drug77	-7.7

drug609	-7.7	drug215	-7.9	drug117	-7.7
drug635	-7.7	drug216	-7.9	drug168	-7.7
drug636	-7.7	drug244	-7.9	drug170	-7.7
drug640	-7.7	drug312	-7.9	drug199	-7.7
drug656	-7.7	drug319	-7.9	drug216	-7.7
drug662	-7.7	drug338	-7.9	drug230	-7.7
drug668	-7.7	drug343	-7.9	drug247	-7.7
drug677	-7.7	drug361	-7.9	drug251	-7.7
drug698	-7.7	drug365	-7.9	drug288	-7.7
drug702	-7.7	drug449	-7.9	drug290	-7.7
drug732	-7.7	drug453	-7.9	drug335	-7.7
drug753	-7.7	drug479	-7.9	drug354	-7.7
drug780	-7.7	drug487	-7.9	drug363	-7.7
drug782	-7.7	drug577	-7.9	drug365	-7.7
drug783	-7.7	drug620	-7.9	drug398	-7.7
drug793	-7.7	drug656	-7.9	drug474	-7.7
drug823	-7.7	drug699	-7.9	drug518	-7.7
drug862	-7.7	drug731	-7.9	drug524	-7.7
drug867	-7.7	drug810	-7.9	drug535	-7.7
drug884	-7.7	drug826	-7.9	drug549	-7.7
drug893	-7.7	drug835	-7.9	drug556	-7.7
drug944	-7.7	drug839	-7.9	drug572	-7.7
drug955	-7.7	drug929	-7.9	drug573	-7.7
drug958	-7.7	drug1085	-7.9	drug577	-7.7
drug976	-7.7	drug1136	-7.9	drug579	-7.7
drug985	-7.7	drug1152	-7.9	drug586	-7.7
drug987	-7.7	drug1161	-7.9	drug634	-7.7
drug1033	-7.7	drug1165	-7.9	drug647	-7.7
drug1040	-7.7	drug1169	-7.9	drug666	-7.7
drug1050	-7.7	drug1179	-7.9	drug721	-7.7
drug1102	-7.7	drug1198	-7.9	drug741	-7.7
drug1115	-7.7	drug1278	-7.9	drug764	-7.7
drug1119	-7.7	drug1292	-7.9	drug778	-7.7
drug1126	-7.7	drug1310	-7.9	drug819	-7.7
drug1132	-7.7	drug1317	-7.9	drug824	-7.7
drug1143	-7.7	drug1327	-7.9	drug836	-7.7
drug1162	-7.7	drug1377	-7.9	drug851	-7.7
drug1175	-7.7	drug1397	-7.9	drug901	-7.7
drug1234	-7.7	drug1410	-7.9	drug920	-7.7
drug1239	-7.7	drug1417	-7.9	drug974	-7.7
drug1262	-7.7	drug1433	-7.9	drug1030	-7.7
drug1298	-7.7	drug1453	-7.9	drug1061	-7.7
drug1299	-7.7	drug1457	-7.9	drug1126	-7.7
drug1320	-7.7	drug1461	-7.9	drug1140	-7.7
drug1331	-7.7	drug1469	-7.9	drug1156	-7.7
drug1352	-7.7	drug62	-7.8	drug1165	-7.7
drug1382	-7.7	drug80	-7.8	drug1191	-7.7

drug1403	-7.7	drug238	-7.8	drug1192	-7.7
drug1405	-7.7	drug290	-7.8	drug1214	-7.7
drug1425	-7.7	drug305	-7.8	drug1257	-7.7
drug1433	-7.7	drug307	-7.8	drug1283	-7.7
drug1458	-7.7	drug341	-7.8	drug1292	-7.7
drug1484	-7.7	drug417	-7.8	drug1295	-7.7
drug77	-7.6	drug446	-7.8	drug1331	-7.7
drug85	-7.6	drug451	-7.8	drug1337	-7.7
drug95	-7.6	drug469	-7.8	drug1364	-7.7
drug140	-7.6	drug485	-7.8	drug1366	-7.7
drug141	-7.6	drug524	-7.8	drug1369	-7.7
drug183	-7.6	drug526	-7.8	drug1385	-7.7
drug208	-7.6	drug534	-7.8	drug1386	-7.7
drug212	-7.6	drug548	-7.8	drug1423	-7.7
drug230	-7.6	drug549	-7.8	drug1427	-7.7
drug231	-7.6	drug558	-7.8	drug1429	-7.7
drug232	-7.6	drug623	-7.8	drug1466	-7.7
drug257	-7.6	drug697	-7.8	drug1478	-7.7
drug321	-7.6	drug698	-7.8	drug1488	-7.7
drug336	-7.6	drug737	-7.8	drug43	-7.6
drug359	-7.6	drug740	-7.8	drug56	-7.6
drug363	-7.6	drug776	-7.8	drug119	-7.6
drug368	-7.6	drug834	-7.8	drug136	-7.6
drug407	-7.6	drug878	-7.8	drug189	-7.6
drug458	-7.6	drug897	-7.8	drug245	-7.6
drug472	-7.6	drug899	-7.8	drug257	-7.6
drug616	-7.6	drug904	-7.8	drug265	-7.6
drug644	-7.6	drug917	-7.8	drug279	-7.6
drug645	-7.6	drug1014	-7.8	drug301	-7.6
drug655	-7.6	drug1042	-7.8	drug374	-7.6
drug661	-7.6	drug1051	-7.8	drug375	-7.6
drug669	-7.6	drug1094	-7.8	drug420	-7.6
drug681	-7.6	drug1116	-7.8	drug462	-7.6
drug701	-7.6	drug1137	-7.8	drug499	-7.6
drug768	-7.6	drug1153	-7.8	drug548	-7.6
drug781	-7.6	drug1189	-7.8	drug576	-7.6
drug834	-7.6	drug1193	-7.8	drug609	-7.6
drug866	-7.6	drug1196	-7.8	drug650	-7.6
drug885	-7.6	drug1247	-7.8	drug652	-7.6
drug888	-7.6	drug1252	-7.8	drug654	-7.6
drug910	-7.6	drug1254	-7.8	drug667	-7.6
drug916	-7.6	drug1261	-7.8	drug715	-7.6
drug919	-7.6	drug1266	-7.8	drug719	-7.6
drug941	-7.6	drug1299	-7.8	drug772	-7.6
drug975	-7.6	drug1313	-7.8	drug791	-7.6
drug980	-7.6	drug1382	-7.8	drug860	-7.6
drug996	-7.6	drug1412	-7.8	drug865	-7.6

drug1019	-7.6	drug1446	-7.8	drug871	-7.6
drug1042	-7.6	drug1474	-7.8	drug886	-7.6
drug1047	-7.6	drug12	-7.7	drug888	-7.6
drug1062	-7.6	drug43	-7.7	drug911	-7.6
drug1094	-7.6	drug141	-7.7	drug959	-7.6
drug1109	-7.6	drug183	-7.7	drug976	-7.6
drug1117	-7.6	drug192	-7.7	drug1019	-7.6
drug1120	-7.6	drug259	-7.7	drug1039	-7.6
drug1129	-7.6	drug298	-7.7	drug1062	-7.6
drug1137	-7.6	drug302	-7.7	drug1077	-7.6
drug1138	-7.6	drug322	-7.7	drug1118	-7.6
drug1199	-7.6	drug366	-7.7	drug1133	-7.6
drug1200	-7.6	drug408	-7.7	drug1139	-7.6
drug1203	-7.6	drug419	-7.7	drug1164	-7.6
drug1211	-7.6	drug473	-7.7	drug1181	-7.6
drug1251	-7.6	drug488	-7.7	drug1209	-7.6
drug1252	-7.6	drug506	-7.7	drug1227	-7.6
drug1256	-7.6	drug510	-7.7	drug1275	-7.6
drug1261	-7.6	drug519	-7.7	drug1317	-7.6
drug1264	-7.6	drug550	-7.7	drug1336	-7.6
drug1296	-7.6	drug576	-7.7	drug1353	-7.6
drug1311	-7.6	drug579	-7.7	drug1373	-7.6
drug1330	-7.6	drug667	-7.7	drug1404	-7.6
drug1332	-7.6	drug668	-7.7	drug1409	-7.6
drug1346	-7.6	drug681	-7.7	drug1457	-7.6
drug1353	-7.6	drug682	-7.7	drug1465	-7.6
drug1366	-7.6	drug693	-7.7	drug59	-7.5
drug1372	-7.6	drug728	-7.7	drug129	-7.5
drug1373	-7.6	drug765	-7.7	drug141	-7.5
drug1423	-7.6	drug865	-7.7	drug264	-7.5
drug1446	-7.6	drug884	-7.7	drug274	-7.5
drug1459	-7.6	drug916	-7.7	drug286	-7.5
drug1470	-7.6	drug955	-7.7	drug287	-7.5
drug1473	-7.6	drug996	-7.7	drug293	-7.5
drug1474	-7.6	drug1002	-7.7	drug312	-7.5
drug1491	-7.6	drug1092	-7.7	drug314	-7.5
drug1498	-7.6	drug1142	-7.7	drug318	-7.5
drug60	-7.5	drug1150	-7.7	drug346	-7.5
drug83	-7.5	drug1205	-7.7	drug356	-7.5
drug84	-7.5	drug1208	-7.7	drug364	-7.5
drug94	-7.5	drug1280	-7.7	drug389	-7.5
drug97	-7.5	drug1293	-7.7	drug476	-7.5
drug109	-7.5	drug1333	-7.7	drug498	-7.5
drug129	-7.5	drug1424	-7.7	drug550	-7.5
drug265	-7.5	drug1431	-7.7	drug583	-7.5
drug272	-7.5	drug1458	-7.7	drug592	-7.5
drug275	-7.5	drug47	-7.6	drug604	-7.5

drug288	-7.5	drug53	-7.6	drug674	-7.5
drug301	-7.5	drug90	-7.6	drug691	-7.5
drug362	-7.5	drug103	-7.6	drug748	-7.5
drug373	-7.5	drug168	-7.6	drug752	-7.5
drug384	-7.5	drug187	-7.6	drug755	-7.5
drug397	-7.5	drug201	-7.6	drug761	-7.5
drug473	-7.5	drug228	-7.6	drug825	-7.5
drug499	-7.5	drug236	-7.6	drug827	-7.5
drug551	-7.5	drug303	-7.6	drug837	-7.5
drug563	-7.5	drug328	-7.6	drug881	-7.5
drug565	-7.5	drug367	-7.6	drug941	-7.5
drug585	-7.5	drug394	-7.6	drug943	-7.5
drug588	-7.5	drug401	-7.6	drug957	-7.5
drug602	-7.5	drug403	-7.6	drug968	-7.5
drug631	-7.5	drug574	-7.6	drug1008	-7.5
drug687	-7.5	drug602	-7.6	drug1020	-7.5
drug699	-7.5	drug617	-7.6	drug1027	-7.5
drug743	-7.5	drug630	-7.6	drug1031	-7.5
drug798	-7.5	drug648	-7.6	drug1040	-7.5
drug827	-7.5	drug659	-7.6	drug1048	-7.5
drug868	-7.5	drug684	-7.6	drug1070	-7.5
drug922	-7.5	drug717	-7.6	drug1138	-7.5
drug924	-7.5	drug746	-7.6	drug1148	-7.5
drug933	-7.5	drug764	-7.6	drug1161	-7.5
drug949	-7.5	drug773	-7.6	drug1190	-7.5
drug964	-7.5	drug880	-7.6	drug1226	-7.5
drug981	-7.5	drug900	-7.6	drug1231	-7.5
drug984	-7.5	drug911	-7.6	drug1238	-7.5
drug1000	-7.5	drug934	-7.6	drug1247	-7.5
drug1001	-7.5	drug946	-7.6	drug1251	-7.5
drug1031	-7.5	drug1010	-7.6	drug1291	-7.5
drug1048	-7.5	drug1037	-7.6	drug1312	-7.5
drug1049	-7.5	drug1041	-7.6	drug1349	-7.5
drug1118	-7.5	drug1045	-7.6	drug1363	-7.5
drug1127	-7.5	drug1057	-7.6	drug1396	-7.5
drug1144	-7.5	drug1066	-7.6	drug1401	-7.5
drug1191	-7.5	drug1078	-7.6	drug1446	-7.5
drug1206	-7.5	drug1131	-7.6	drug1477	-7.5
drug1237	-7.5	drug1138	-7.6	drug1484	-7.5
drug1250	-7.5	drug1143	-7.6	drug12	-7.4
drug1254	-7.5	drug1217	-7.6	drug28	-7.4
drug1275	-7.5	drug1226	-7.6	drug47	-7.4
drug1289	-7.5	drug1231	-7.6	drug53	-7.4
drug1295	-7.5	drug1238	-7.6	drug55	-7.4
drug1300	-7.5	drug1250	-7.6	drug74	-7.4
drug1302	-7.5	drug1311	-7.6	drug79	-7.4
drug1325	-7.5	drug1344	-7.6	drug95	-7.4

drug1345	-7.5	drug1358	-7.6	drug114	-7.4
drug1385	-7.5	drug1369	-7.6	drug115	-7.4
drug1407	-7.5	drug1378	-7.6	drug120	-7.4
drug1409	-7.5	drug1396	-7.6	drug135	-7.4
drug1417	-7.5	drug1401	-7.6	drug198	-7.4
drug1469	-7.5	drug1411	-7.6	drug208	-7.4
drug1475	-7.5	drug1418	-7.6	drug228	-7.4
drug28	-7.4	drug1438	-7.6	drug259	-7.4
drug56	-7.4	drug1459	-7.6	drug268	-7.4
drug73	-7.4	drug1499	-7.6	drug276	-7.4
drug86	-7.4	drug27	-7.5	drug277	-7.4
drug130	-7.4	drug33	-7.5	drug304	-7.4
drug198	-7.4	drug78	-7.5	drug317	-7.4
drug206	-7.4	drug125	-7.5	drug326	-7.4
drug248	-7.4	drug129	-7.5	drug329	-7.4
drug305	-7.4	drug138	-7.5	drug333	-7.4
drug306	-7.4	drug159	-7.5	drug362	-7.4
drug317	-7.4	drug200	-7.5	drug387	-7.4
drug327	-7.4	drug281	-7.5	drug422	-7.4
drug334	-7.4	drug395	-7.5	drug495	-7.4
drug364	-7.4	drug406	-7.5	drug504	-7.4
drug374	-7.4	drug544	-7.5	drug564	-7.4
drug375	-7.4	drug601	-7.5	drug567	-7.4
drug418	-7.4	drug614	-7.5	drug587	-7.4
drug435	-7.4	drug650	-7.5	drug619	-7.4
drug457	-7.4	drug709	-7.5	drug637	-7.4
drug469	-7.4	drug752	-7.5	drug644	-7.4
drug470	-7.4	drug759	-7.5	drug658	-7.4
drug503	-7.4	drug761	-7.5	drug661	-7.4
drug519	-7.4	drug763	-7.5	drug696	-7.4
drug574	-7.4	drug784	-7.5	drug698	-7.4
drug601	-7.4	drug820	-7.5	drug727	-7.4
drug604	-7.4	drug821	-7.5	drug768	-7.4
drug629	-7.4	drug831	-7.5	drug769	-7.4
drug637	-7.4	drug860	-7.5	drug773	-7.4
drug726	-7.4	drug928	-7.5	drug784	-7.4
drug756	-7.4	drug1019	-7.5	drug826	-7.4
drug795	-7.4	drug1032	-7.5	drug843	-7.4
drug799	-7.4	drug1067	-7.5	drug850	-7.4
drug801	-7.4	drug1074	-7.5	drug870	-7.4
drug809	-7.4	drug1075	-7.5	drug890	-7.4
drug848	-7.4	drug1103	-7.5	drug899	-7.4
drug872	-7.4	drug1159	-7.5	drug927	-7.4
drug887	-7.4	drug1170	-7.5	drug958	-7.4
drug952	-7.4	drug1195	-7.5	drug972	-7.4
drug959	-7.4	drug1199	-7.5	drug1022	-7.4
drug968	-7.4	drug1200	-7.5	drug1049	-7.4

drug1027	-7.4	drug1210	-7.5	drug1071	-7.4
drug1030	-7.4	drug1284	-7.5	drug1152	-7.4
drug1037	-7.4	drug1298	-7.5	drug1207	-7.4
drug1045	-7.4	drug1300	-7.5	drug1212	-7.4
drug1064	-7.4	drug1302	-7.5	drug1217	-7.4
drug1122	-7.4	drug1326	-7.5	drug1219	-7.4
drug1139	-7.4	drug1354	-7.5	drug1221	-7.4
drug1141	-7.4	drug1364	-7.5	drug1224	-7.4
drug1145	-7.4	drug1366	-7.5	drug1243	-7.4
drug1160	-7.4	drug1405	-7.5	drug1256	-7.4
drug1167	-7.4	drug1449	-7.5	drug1281	-7.4
drug1170	-7.4	drug1466	-7.5	drug1286	-7.4
drug1179	-7.4	drug1475	-7.5	drug1352	-7.4
drug1267	-7.4	drug20	-7.4	drug1358	-7.4
drug1278	-7.4	drug30	-7.4	drug1374	-7.4
drug1292	-7.4	drug60	-7.4	drug1403	-7.4
drug1303	-7.4	drug136	-7.4	drug1405	-7.4
drug1322	-7.4	drug157	-7.4	drug1417	-7.4
drug1337	-7.4	drug232	-7.4	drug1454	-7.4
drug1347	-7.4	drug247	-7.4	drug23	-7.3
drug1350	-7.4	drug256	-7.4	drug58	-7.3
drug1369	-7.4	drug272	-7.4	drug85	-7.3
drug1374	-7.4	drug293	-7.4	drug187	-7.3
drug1397	-7.4	drug304	-7.4	drug206	-7.3
drug1404	-7.4	drug314	-7.4	drug273	-7.3
drug1418	-7.4	drug316	-7.4	drug292	-7.3
drug1440	-7.4	drug317	-7.4	drug305	-7.3
drug1444	-7.4	drug371	-7.4	drug306	-7.3
drug1452	-7.4	drug457	-7.4	drug325	-7.3
drug1477	-7.4	drug461	-7.4	drug334	-7.3
drug23	-7.3	drug535	-7.4	drug355	-7.3
drug75	-7.3	drug613	-7.4	drug401	-7.3
drug168	-7.3	drug629	-7.4	drug427	-7.3
drug228	-7.3	drug645	-7.4	drug442	-7.3
drug251	-7.3	drug660	-7.4	drug457	-7.3
drug279	-7.3	drug663	-7.4	drug472	-7.3
drug281	-7.3	drug719	-7.4	drug558	-7.3
drug287	-7.3	drug739	-7.4	drug568	-7.3
drug319	-7.3	drug745	-7.4	drug589	-7.3
drug338	-7.3	drug748	-7.4	drug598	-7.3
drug348	-7.3	drug806	-7.4	drug599	-7.3
drug361	-7.3	drug837	-7.4	drug671	-7.3
drug462	-7.3	drug875	-7.4	drug677	-7.3
drug500	-7.3	drug883	-7.4	drug713	-7.3
drug517	-7.3	drug962	-7.4	drug722	-7.3
drug531	-7.3	drug1044	-7.4	drug737	-7.3
drug535	-7.3	drug1140	-7.4	drug790	-7.3

drug537	-7.3	drug1145	-7.4	drug795	-7.3
drug576	-7.3	drug1148	-7.4	drug808	-7.3
drug583	-7.3	drug1172	-7.4	drug810	-7.3
drug665	-7.3	drug1212	-7.4	drug813	-7.3
drug666	-7.3	drug1213	-7.4	drug849	-7.3
drug667	-7.3	drug1282	-7.4	drug867	-7.3
drug729	-7.3	drug1297	-7.4	drug868	-7.3
drug758	-7.3	drug1384	-7.4	drug895	-7.3
drug761	-7.3	drug1422	-7.4	drug900	-7.3
drug763	-7.3	drug1444	-7.4	drug908	-7.3
drug778	-7.3	drug1452	-7.4	drug912	-7.3
drug784	-7.3	drug1455	-7.4	drug947	-7.3
drug788	-7.3	drug1472	-7.4	drug1000	-7.3
drug806	-7.3	drug1485	-7.4	drug1002	-7.3
drug889	-7.3	drug3	-7.3	drug1032	-7.3
drug896	-7.3	drug8	-7.3	drug1036	-7.3
drug913	-7.3	drug11	-7.3	drug1045	-7.3
drug920	-7.3	drug14	-7.3	drug1047	-7.3
drug927	-7.3	drug75	-7.3	drug1065	-7.3
drug940	-7.3	drug137	-7.3	drug1072	-7.3
drug982	-7.3	drug156	-7.3	drug1078	-7.3
drug1010	-7.3	drug162	-7.3	drug1094	-7.3
drug1011	-7.3	drug199	-7.3	drug1103	-7.3
drug1015	-7.3	drug217	-7.3	drug1109	-7.3
drug1025	-7.3	drug364	-7.3	drug1142	-7.3
drug1036	-7.3	drug382	-7.3	drug1149	-7.3
drug1061	-7.3	drug399	-7.3	drug1167	-7.3
drug1159	-7.3	drug455	-7.3	drug1210	-7.3
drug1164	-7.3	drug483	-7.3	drug1245	-7.3
drug1192	-7.3	drug522	-7.3	drug1262	-7.3
drug1214	-7.3	drug527	-7.3	drug1290	-7.3
drug1247	-7.3	drug545	-7.3	drug1327	-7.3
drug1253	-7.3	drug567	-7.3	drug1377	-7.3
drug1265	-7.3	drug587	-7.3	drug1382	-7.3
drug1283	-7.3	drug628	-7.3	drug1393	-7.3
drug1291	-7.3	drug649	-7.3	drug1398	-7.3
drug1293	-7.3	drug671	-7.3	drug1415	-7.3
drug1324	-7.3	drug683	-7.3	drug1426	-7.3
drug1349	-7.3	drug742	-7.3	drug1447	-7.3
drug1354	-7.3	drug743	-7.3	drug1452	-7.3
drug1377	-7.3	drug750	-7.3	drug1472	-7.3
drug1398	-7.3	drug756	-7.3	drug1473	-7.3
drug1436	-7.3	drug777	-7.3	drug1499	-7.3
drug1445	-7.3	drug788	-7.3	drug49	-7.2
drug1449	-7.3	drug794	-7.3	drug84	-7.2
drug9	-7.2	drug805	-7.3	drug200	-7.2
drug43	-7.2	drug814	-7.3	drug303	-7.2

drug70	-7.2	drug824	-7.3	drug316	-7.2
drug74	-7.2	drug825	-7.3	drug330	-7.2
drug90	-7.2	drug840	-7.3	drug360	-7.2
drug114	-7.2	drug845	-7.3	drug473	-7.2
drug121	-7.2	drug849	-7.3	drug509	-7.2
drug137	-7.2	drug850	-7.3	drug516	-7.2
drug143	-7.2	drug893	-7.3	drug607	-7.2
drug165	-7.2	drug931	-7.3	drug645	-7.2
drug210	-7.2	drug941	-7.3	drug663	-7.2
drug243	-7.2	drug953	-7.3	drug682	-7.2
drug262	-7.2	drug969	-7.3	drug693	-7.2
drug297	-7.2	drug1026	-7.3	drug759	-7.2
drug325	-7.2	drug1048	-7.3	drug783	-7.2
drug344	-7.2	drug1059	-7.3	drug787	-7.2
drug376	-7.2	drug1070	-7.3	drug788	-7.2
drug398	-7.2	drug1107	-7.3	drug835	-7.2
drug467	-7.2	drug1134	-7.3	drug845	-7.2
drug477	-7.2	drug1147	-7.3	drug872	-7.2
drug498	-7.2	drug1164	-7.3	drug889	-7.2
drug521	-7.2	drug1173	-7.3	drug893	-7.2
drug540	-7.2	drug1190	-7.3	drug897	-7.2
drug575	-7.2	drug1211	-7.3	drug921	-7.2
drug592	-7.2	drug1222	-7.3	drug922	-7.2
drug641	-7.2	drug1296	-7.3	drug954	-7.2
drug650	-7.2	drug1336	-7.3	drug960	-7.2
drug673	-7.2	drug1476	-7.3	drug973	-7.2
drug706	-7.2	drug70	-7.2	drug990	-7.2
drug719	-7.2	drug96	-7.2	drug1001	-7.2
drug751	-7.2	drug120	-7.2	drug1038	-7.2
drug790	-7.2	drug181	-7.2	drug1041	-7.2
drug808	-7.2	drug213	-7.2	drug1044	-7.2
drug812	-7.2	drug224	-7.2	drug1055	-7.2
drug814	-7.2	drug263	-7.2	drug1085	-7.2
drug860	-7.2	drug296	-7.2	drug1131	-7.2
drug865	-7.2	drug299	-7.2	drug1137	-7.2
drug881	-7.2	drug315	-7.2	drug1151	-7.2
drug906	-7.2	drug374	-7.2	drug1159	-7.2
drug914	-7.2	drug389	-7.2	drug1296	-7.2
drug957	-7.2	drug459	-7.2	drug1297	-7.2
drug991	-7.2	drug471	-7.2	drug1300	-7.2
drug1070	-7.2	drug529	-7.2	drug1322	-7.2
drug1103	-7.2	drug531	-7.2	drug1416	-7.2
drug1107	-7.2	drug553	-7.2	drug1455	-7.2
drug1151	-7.2	drug631	-7.2	drug1470	-7.2
drug1163	-7.2	drug636	-7.2	drug1485	-7.2
drug1181	-7.2	drug680	-7.2	drug86	-7.1
drug1220	-7.2	drug747	-7.2	drug93	-7.1

drug1223	-7.2	drug751	-7.2	drug231	-7.1
drug1226	-7.2	drug786	-7.2	drug232	-7.1
drug1238	-7.2	drug791	-7.2	drug242	-7.1
drug1276	-7.2	drug792	-7.2	drug248	-7.1
drug1301	-7.2	drug798	-7.2	drug256	-7.1
drug1318	-7.2	drug807	-7.2	drug322	-7.1
drug1348	-7.2	drug822	-7.2	drug370	-7.1
drug1375	-7.2	drug827	-7.2	drug423	-7.1
drug1386	-7.2	drug833	-7.2	drug492	-7.1
drug1393	-7.2	drug843	-7.2	drug521	-7.1
drug1453	-7.2	drug861	-7.2	drug538	-7.1
drug1476	-7.2	drug885	-7.2	drug547	-7.1
drug1492	-7.2	drug895	-7.2	drug574	-7.1
drug1495	-7.2	drug1004	-7.2	drug575	-7.1
drug33	-7.1	drug1015	-7.2	drug582	-7.1
drug64	-7.1	drug1068	-7.2	drug649	-7.1
drug144	-7.1	drug1105	-7.2	drug687	-7.1
drug153	-7.1	drug1146	-7.2	drug697	-7.1
drug273	-7.1	drug1203	-7.2	drug699	-7.1
drug318	-7.1	drug1245	-7.2	drug726	-7.1
drug329	-7.1	drug1304	-7.2	drug728	-7.1
drug346	-7.1	drug1319	-7.2	drug756	-7.1
drug370	-7.1	drug1359	-7.2	drug807	-7.1
drug382	-7.1	drug1398	-7.2	drug840	-7.1
drug420	-7.1	drug1409	-7.2	drug887	-7.1
drug442	-7.1	drug1413	-7.2	drug896	-7.1
drug518	-7.1	drug1415	-7.2	drug933	-7.1
drug529	-7.1	drug1429	-7.2	drug934	-7.1
drug545	-7.1	drug1445	-7.2	drug970	-7.1
drug550	-7.1	drug31	-7.1	drug975	-7.1
drug573	-7.1	drug38	-7.1	drug985	-7.1
drug587	-7.1	drug76	-7.1	drug1012	-7.1
drug589	-7.1	drug242	-7.1	drug1035	-7.1
drug613	-7.1	drug265	-7.1	drug1037	-7.1
drug658	-7.1	drug306	-7.1	drug1091	-7.1
drug680	-7.1	drug355	-7.1	drug1213	-7.1
drug685	-7.1	drug358	-7.1	drug1253	-7.1
drug696	-7.1	drug359	-7.1	drug1276	-7.1
drug737	-7.1	drug396	-7.1	drug1277	-7.1
drug745	-7.1	drug428	-7.1	drug1278	-7.1
drug764	-7.1	drug538	-7.1	drug1298	-7.1
drug773	-7.1	drug547	-7.1	drug1332	-7.1
drug791	-7.1	drug564	-7.1	drug1437	-7.1
drug805	-7.1	drug582	-7.1	drug1481	-7.1
drug810	-7.1	drug619	-7.1	drug11	-7
drug849	-7.1	drug644	-7.1	drug70	-7
drug850	-7.1	drug688	-7.1	drug215	-7

drug869	-7.1	drug705	-7.1	drug238	-7
drug895	-7.1	drug722	-7.1	drug244	-7
drug899	-7.1	drug778	-7.1	drug285	-7
drug911	-7.1	drug812	-7.1	drug376	-7
drug943	-7.1	drug854	-7.1	drug390	-7
drug945	-7.1	drug855	-7.1	drug399	-7
drug965	-7.1	drug863	-7.1	drug418	-7
drug974	-7.1	drug869	-7.1	drug435	-7
drug1008	-7.1	drug907	-7.1	drug503	-7
drug1016	-7.1	drug991	-7.1	drug553	-7
drug1035	-7.1	drug1049	-7.1	drug610	-7
drug1066	-7.1	drug1090	-7.1	drug680	-7
drug1083	-7.1	drug1108	-7.1	drug681	-7
drug1085	-7.1	drug1209	-7.1	drug684	-7
drug1152	-7.1	drug1244	-7.1	drug706	-7
drug1158	-7.1	drug1285	-7.1	drug711	-7
drug1161	-7.1	drug1301	-7.1	drug782	-7
drug1197	-7.1	drug1338	-7.1	drug789	-7
drug1205	-7.1	drug1345	-7.1	drug812	-7
drug1255	-7.1	drug1379	-7.1	drug822	-7
drug1260	-7.1	drug1387	-7.1	drug831	-7
drug1277	-7.1	drug1400	-7.1	drug839	-7
drug1290	-7.1	drug1416	-7.1	drug915	-7
drug1355	-7.1	drug6	-7	drug932	-7
drug1362	-7.1	drug65	-7	drug967	-7
drug1363	-7.1	drug72	-7	drug1011	-7
drug1419	-7.1	drug86	-7	drug1015	-7
drug1455	-7.1	drug135	-7	drug1025	-7
drug1461	-7.1	drug142	-7	drug1046	-7
drug1472	-7.1	drug144	-7	drug1075	-7
drug1499	-7.1	drug153	-7	drug1107	-7
drug22	-7	drug218	-7	drug1112	-7
drug38	-7	drug223	-7	drug1120	-7
drug146	-7	drug227	-7	drug1293	-7
drug175	-7	drug280	-7	drug1299	-7
drug181	-7	drug331	-7	drug1303	-7
drug214	-7	drug435	-7	drug1318	-7
drug218	-7	drug603	-7	drug1365	-7
drug227	-7	drug612	-7	drug1375	-7
drug241	-7	drug621	-7	drug1383	-7
drug276	-7	drug633	-7	drug1388	-7
drug277	-7	drug686	-7	drug1418	-7
drug290	-7	drug708	-7	drug1438	-7
drug304	-7	drug755	-7	drug1461	-7
drug383	-7	drug780	-7	drug94	-6.9
drug395	-7	drug782	-7	drug218	-6.9
drug459	-7	drug838	-7	drug299	-6.9

drug530	-7	drug862	-7	drug307	-6.9
drug559	-7	drug872	-7	drug336	-6.9
drug630	-7	drug876	-7	drug371	-6.9
drug659	-7	drug890	-7	drug382	-6.9
drug660	-7	drug908	-7	drug397	-6.9
drug710	-7	drug942	-7	drug529	-6.9
drug759	-7	drug943	-7	drug621	-6.9
drug807	-7	drug992	-7	drug635	-6.9
drug824	-7	drug1009	-7	drug659	-6.9
drug839	-7	drug1016	-7	drug688	-6.9
drug931	-7	drug1091	-7	drug712	-6.9
drug934	-7	drug1095	-7	drug742	-6.9
drug1056	-7	drug1133	-7	drug781	-6.9
drug1059	-7	drug1135	-7	drug792	-6.9
drug1088	-7	drug1181	-7	drug806	-6.9
drug1090	-7	drug1265	-7	drug811	-6.9
drug1123	-7	drug1376	-7	drug880	-6.9
drug1142	-7	drug1395	-7	drug930	-6.9
drug1148	-7	drug1414	-7	drug946	-6.9
drug1165	-7	drug1440	-7	drug965	-6.9
drug1216	-7	drug1451	-7	drug969	-6.9
drug1217	-7	drug1463	-7	drug1009	-6.9
drug1219	-7	drug1482	-7	drug1014	-6.9
drug1229	-7	drug1495	-7	drug1059	-6.9
drug1242	-7	drug35	-6.9	drug1087	-6.9
drug1284	-7	drug145	-6.9	drug1088	-6.9
drug1378	-7	drug146	-6.9	drug1089	-6.9
drug1390	-7	drug255	-6.9	drug1108	-6.9
drug1396	-7	drug274	-6.9	drug1170	-6.9
drug1438	-7	drug292	-6.9	drug1173	-6.9
drug1450	-7	drug330	-6.9	drug1203	-6.9
drug1482	-7	drug356	-6.9	drug1206	-6.9
drug1483	-7	drug397	-6.9	drug1334	-6.9
drug1488	-7	drug445	-6.9	drug1360	-6.9
drug11	-6.9	drug477	-6.9	drug1445	-6.9
drug19	-6.9	drug490	-6.9	drug1449	-6.9
drug30	-6.9	drug530	-6.9	drug1482	-6.9
drug32	-6.9	drug559	-6.9	drug1498	-6.9
drug176	-6.9	drug589	-6.9	drug3	-6.8
drug213	-6.9	drug610	-6.9	drug8	-6.8
drug217	-6.9	drug639	-6.9	drug22	-6.8
drug221	-6.9	drug642	-6.9	drug30	-6.8
drug278	-6.9	drug673	-6.9	drug90	-6.8
drug285	-6.9	drug700	-6.9	drug97	-6.8
drug296	-6.9	drug720	-6.9	drug144	-6.8
drug298	-6.9	drug736	-6.9	drug145	-6.8
drug299	-6.9	drug804	-6.9	drug157	-6.8

drug300	-6.9	drug909	-6.9	drug302	-6.8
drug312	-6.9	drug951	-6.9	drug338	-6.8
drug509	-6.9	drug960	-6.9	drug452	-6.8
drug516	-6.9	drug966	-6.9	drug483	-6.8
drug524	-6.9	drug1011	-6.9	drug522	-6.8
drug538	-6.9	drug1022	-6.9	drug526	-6.8
drug544	-6.9	drug1024	-6.9	drug559	-6.8
drug548	-6.9	drug1035	-6.9	drug601	-6.8
drug643	-6.9	drug1047	-6.9	drug636	-6.8
drug649	-6.9	drug1086	-6.9	drug655	-6.8
drug689	-6.9	drug1118	-6.9	drug685	-6.8
drug690	-6.9	drug1123	-6.9	drug707	-6.8
drug712	-6.9	drug1128	-6.9	drug743	-6.8
drug752	-6.9	drug1149	-6.9	drug815	-6.8
drug811	-6.9	drug1163	-6.9	drug913	-6.8
drug816	-6.9	drug1197	-6.9	drug914	-6.8
drug825	-6.9	drug1221	-6.9	drug945	-6.8
drug826	-6.9	drug1223	-6.9	drug962	-6.8
drug845	-6.9	drug1229	-6.9	drug979	-6.8
drug900	-6.9	drug1286	-6.9	drug983	-6.8
drug962	-6.9	drug1312	-6.9	drug991	-6.8
drug1004	-6.9	drug1346	-6.9	drug1034	-6.8
drug1071	-6.9	drug1361	-6.9	drug1147	-6.8
drug1072	-6.9	drug1389	-6.9	drug1172	-6.8
drug1087	-6.9	drug1391	-6.9	drug1205	-6.8
drug1106	-6.9	drug1426	-6.9	drug1237	-6.8
drug1112	-6.9	drug1447	-6.9	drug1260	-6.8
drug1124	-6.9	drug1448	-6.9	drug1265	-6.8
drug1135	-6.9	drug1462	-6.9	drug1285	-6.8
drug1224	-6.9	drug1464	-6.9	drug1301	-6.8
drug1259	-6.9	drug1487	-6.9	drug1329	-6.8
drug1342	-6.9	drug1497	-6.9	drug1390	-6.8
drug1360	-6.9	drug49	-6.8	drug1450	-6.8
drug1364	-6.9	drug64	-6.8	drug1453	-6.8
drug1383	-6.9	drug108	-6.8	drug1462	-6.8
drug1388	-6.9	drug130	-6.8	drug1483	-6.8
drug1392	-6.9	drug143	-6.8	drug6	-6.7
drug1481	-6.9	drug175	-6.8	drug7	-6.7
drug8	-6.8	drug318	-6.8	drug45	-6.7
drug35	-6.8	drug329	-6.8	drug91	-6.7
drug49	-6.8	drug336	-6.8	drug96	-6.7
drug91	-6.8	drug390	-6.8	drug154	-6.7
drug120	-6.8	drug500	-6.8	drug159	-6.7
drug156	-6.8	drug514	-6.8	drug165	-6.7
drug157	-6.8	drug566	-6.8	drug177	-6.7
drug167	-6.8	drug568	-6.8	drug271	-6.7
drug215	-6.8	drug570	-6.8	drug294	-6.7

drug223	-6.8	drug571	-6.8	drug321	-6.7
drug244	-6.8	drug635	-6.8	drug348	-6.7
drug280	-6.8	drug675	-6.8	drug358	-6.7
drug314	-6.8	drug690	-6.8	drug412	-6.7
drug315	-6.8	drug707	-6.8	drug454	-6.7
drug354	-6.8	drug715	-6.8	drug542	-6.7
drug360	-6.8	drug797	-6.8	drug565	-6.7
drug389	-6.8	drug813	-6.8	drug570	-6.7
drug423	-6.8	drug913	-6.8	drug739	-6.7
drug433	-6.8	drug914	-6.8	drug763	-6.7
drug483	-6.8	drug933	-6.8	drug785	-6.7
drug490	-6.8	drug982	-6.8	drug786	-6.7
drug514	-6.8	drug984	-6.8	drug798	-6.7
drug525	-6.8	drug988	-6.8	drug805	-6.7
drug553	-6.8	drug1008	-6.8	drug841	-6.7
drug642	-6.8	drug1071	-6.8	drug853	-6.7
drug684	-6.8	drug1096	-6.8	drug854	-6.7
drug693	-6.8	drug1109	-6.8	drug863	-6.7
drug697	-6.8	drug1112	-6.8	drug869	-6.7
drug707	-6.8	drug1130	-6.8	drug916	-6.7
drug711	-6.8	drug1309	-6.8	drug952	-6.7
drug717	-6.8	drug1318	-6.8	drug955	-6.7
drug739	-6.8	drug1329	-6.8	drug1083	-6.7
drug831	-6.8	drug1350	-6.8	drug1096	-6.7
drug840	-6.8	drug1388	-6.8	drug1106	-6.7
drug843	-6.8	drug1393	-6.8	drug1128	-6.7
drug853	-6.8	drug1479	-6.8	drug1135	-6.7
drug863	-6.8	drug1486	-6.8	drug1197	-6.7
drug870	-6.8	drug1498	-6.8	drug1302	-6.7
drug946	-6.8	drug106	-6.7	drug1305	-6.7
drug954	-6.8	drug202	-6.7	drug1338	-6.7
drug961	-6.8	drug262	-6.7	drug1361	-6.7
drug969	-6.8	drug277	-6.7	drug1362	-6.7
drug983	-6.8	drug283	-6.7	drug1392	-6.7
drug1022	-6.8	drug285	-6.7	drug1414	-6.7
drug1046	-6.8	drug297	-6.7	drug1432	-6.7
drug1055	-6.8	drug423	-6.7	drug1476	-6.7
drug1086	-6.8	drug452	-6.7	drug1492	-6.7
drug1089	-6.8	drug468	-6.7	drug31	-6.6
drug1091	-6.8	drug492	-6.7	drug32	-6.6
drug1096	-6.8	drug540	-6.7	drug130	-6.6
drug1149	-6.8	drug541	-6.7	drug280	-6.6
drug1314	-6.8	drug590	-6.7	drug298	-6.6
drug1329	-6.8	drug712	-6.7	drug372	-6.6
drug1338	-6.8	drug725	-6.7	drug459	-6.6
drug1391	-6.8	drug735	-6.7	drug468	-6.6
drug1479	-6.8	drug781	-6.7	drug525	-6.6

drug13	-6.7	drug787	-6.7	drug571	-6.6
drug34	-6.7	drug815	-6.7	drug590	-6.6
drug63	-6.7	drug828	-6.7	drug642	-6.6
drug65	-6.7	drug836	-6.7	drug673	-6.6
drug67	-6.7	drug922	-6.7	drug675	-6.6
drug76	-6.7	drug925	-6.7	drug690	-6.6
drug133	-6.7	drug958	-6.7	drug801	-6.6
drug145	-6.7	drug993	-6.7	drug907	-6.6
drug160	-6.7	drug1000	-6.7	drug909	-6.6
drug162	-6.7	drug1034	-6.7	drug951	-6.6
drug177	-6.7	drug1050	-6.7	drug966	-6.6
drug205	-6.7	drug1201	-6.7	drug982	-6.6
drug283	-6.7	drug1334	-6.7	drug984	-6.6
drug284	-6.7	drug1342	-6.7	drug1056	-6.6
drug293	-6.7	drug1363	-6.7	drug1058	-6.6
drug322	-6.7	drug1390	-6.7	drug1068	-6.6
drug401	-6.7	drug1450	-6.7	drug1074	-6.6
drug412	-6.7	drug1481	-6.7	drug1123	-6.6
drug427	-6.7	drug19	-6.6	drug1124	-6.6
drug452	-6.7	drug67	-6.6	drug1130	-6.6
drug455	-6.7	drug194	-6.6	drug1146	-6.6
drug526	-6.7	drug271	-6.6	drug1162	-6.6
drug755	-6.7	drug357	-6.6	drug1163	-6.6
drug792	-6.7	drug360	-6.6	drug1235	-6.6
drug796	-6.7	drug372	-6.6	drug1239	-6.6
drug804	-6.7	drug454	-6.6	drug1259	-6.6
drug828	-6.7	drug528	-6.6	drug1326	-6.6
drug858	-6.7	drug569	-6.6	drug1378	-6.6
drug923	-6.7	drug665	-6.6	drug1389	-6.6
drug929	-6.7	drug689	-6.6	drug1391	-6.6
drug960	-6.7	drug713	-6.6	drug1395	-6.6
drug967	-6.7	drug785	-6.6	drug1419	-6.6
drug970	-6.7	drug789	-6.6	drug1451	-6.6
drug1172	-6.7	drug891	-6.6	drug1463	-6.6
drug1210	-6.7	drug892	-6.6	drug1464	-6.6
drug1213	-6.7	drug952	-6.6	drug1479	-6.6
drug1215	-6.7	drug957	-6.6	drug1495	-6.6
drug1243	-6.7	drug1056	-6.6	drug64	-6.5
drug1245	-6.7	drug1072	-6.6	drug162	-6.5
drug1312	-6.7	drug1083	-6.6	drug175	-6.5
drug1395	-6.7	drug1087	-6.6	drug196	-6.5
drug1426	-6.7	drug1088	-6.6	drug202	-6.5
drug1443	-6.7	drug1132	-6.6	drug239	-6.5
drug1451	-6.7	drug1162	-6.6	drug283	-6.5
drug1462	-6.7	drug1167	-6.6	drug431	-6.5
drug1480	-6.7	drug1191	-6.6	drug433	-6.5
drug2	-6.6	drug1243	-6.6	drug530	-6.5

drug4	-6.6	drug1322	-6.6	drug594	-6.5
drug105	-6.6	drug1341	-6.6	drug603	-6.5
drug161	-6.6	drug1374	-6.6	drug780	-6.5
drug196	-6.6	drug1406	-6.6	drug816	-6.5
drug242	-6.6	drug1407	-6.6	drug858	-6.5
drug263	-6.6	drug1483	-6.6	drug1175	-6.5
drug292	-6.6	drug9	-6.5	drug1201	-6.5
drug330	-6.6	drug15	-6.5	drug1223	-6.5
drug454	-6.6	drug32	-6.5	drug1254	-6.5
drug468	-6.6	drug45	-6.5	drug1255	-6.5
drug647	-6.6	drug66	-6.5	drug1284	-6.5
drug664	-6.6	drug105	-6.5	drug1342	-6.5
drug671	-6.6	drug177	-6.5	drug1413	-6.5
drug727	-6.6	drug284	-6.5	drug4	-6.4
drug728	-6.6	drug294	-6.5	drug5	-6.4
drug800	-6.6	drug300	-6.5	drug214	-6.4
drug813	-6.6	drug311	-6.5	drug296	-6.4
drug915	-6.6	drug321	-6.5	drug378	-6.4
drug930	-6.6	drug378	-6.5	drug432	-6.4
drug932	-6.6	drug467	-6.5	drug434	-6.4
drug953	-6.6	drug525	-6.5	drug477	-6.4
drug1044	-6.6	drug542	-6.5	drug531	-6.4
drug1075	-6.6	drug580	-6.5	drug566	-6.4
drug1128	-6.6	drug598	-6.5	drug602	-6.4
drug1130	-6.6	drug647	-6.5	drug670	-6.4
drug1306	-6.6	drug657	-6.5	drug804	-6.4
drug1361	-6.6	drug664	-6.5	drug838	-6.4
drug1389	-6.6	drug685	-6.5	drug862	-6.4
drug1463	-6.6	drug718	-6.5	drug942	-6.4
drug3	-6.5	drug724	-6.5	drug944	-6.4
drug5	-6.5	drug803	-6.5	drug953	-6.4
drug14	-6.5	drug811	-6.5	drug1050	-6.4
drug51	-6.5	drug823	-6.5	drug1086	-6.4
drug108	-6.5	drug879	-6.5	drug1104	-6.4
drug123	-6.5	drug888	-6.5	drug1157	-6.4
drug159	-6.5	drug915	-6.5	drug1158	-6.4
drug240	-6.5	drug944	-6.5	drug1497	-6.4
drug256	-6.5	drug949	-6.5	drug15	-6.3
drug286	-6.5	drug954	-6.5	drug76	-6.3
drug316	-6.5	drug1001	-6.5	drug128	-6.3
drug328	-6.5	drug1227	-6.5	drug137	-6.3
drug422	-6.5	drug1259	-6.5	drug142	-6.3
drug431	-6.5	drug1305	-6.5	drug143	-6.3
drug491	-6.5	drug1316	-6.5	drug181	-6.3
drug492	-6.5	drug1360	-6.5	drug224	-6.3
drug560	-6.5	drug1380	-6.5	drug227	-6.3
drug564	-6.5	drug1392	-6.5	drug254	-6.3

drug598	-6.5	drug1432	-6.5	drug278	-6.3
drug610	-6.5	drug1480	-6.5	drug455	-6.3
drug612	-6.5	drug7	-6.4	drug491	-6.3
drug663	-6.5	drug13	-6.4	drug514	-6.3
drug803	-6.5	drug17	-6.4	drug528	-6.3
drug815	-6.5	drug51	-6.4	drug554	-6.3
drug838	-6.5	drug123	-6.4	drug560	-6.3
drug880	-6.5	drug158	-6.4	drug569	-6.3
drug897	-6.5	drug198	-6.4	drug597	-6.3
drug979	-6.5	drug221	-6.4	drug665	-6.3
drug992	-6.5	drug222	-6.4	drug809	-6.3
drug1014	-6.5	drug225	-6.4	drug823	-6.3
drug1041	-6.5	drug237	-6.4	drug833	-6.3
drug1108	-6.5	drug253	-6.4	drug861	-6.3
drug1173	-6.5	drug264	-6.4	drug903	-6.3
drug1228	-6.5	drug377	-6.4	drug1246	-6.3
drug1297	-6.5	drug434	-6.4	drug1341	-6.3
drug1305	-6.5	drug573	-6.4	drug1440	-6.3
drug1334	-6.5	drug594	-6.4	drug1480	-6.3
drug1464	-6.5	drug790	-6.4	drug9	-6.2
drug17	-6.4	drug809	-6.4	drug13	-6.2
drug45	-6.4	drug842	-6.4	drug19	-6.2
drug96	-6.4	drug923	-6.4	drug35	-6.2
drug106	-6.4	drug967	-6.4	drug65	-6.2
drug116	-6.4	drug983	-6.4	drug105	-6.2
drug128	-6.4	drug1003	-6.4	drug146	-6.2
drug142	-6.4	drug1064	-6.4	drug158	-6.2
drug158	-6.4	drug1124	-6.4	drug222	-6.2
drug274	-6.4	drug1224	-6.4	drug223	-6.2
drug294	-6.4	drug1228	-6.4	drug241	-6.2
drug357	-6.4	drug1246	-6.4	drug262	-6.2
drug434	-6.4	drug4	-6.3	drug284	-6.2
drug541	-6.4	drug5	-6.3	drug300	-6.2
drug542	-6.4	drug37	-6.3	drug328	-6.2
drug566	-6.4	drug116	-6.3	drug349	-6.2
drug594	-6.4	drug122	-6.3	drug413	-6.2
drug654	-6.4	drug128	-6.3	drug475	-6.2
drug708	-6.4	drug133	-6.3	drug490	-6.2
drug724	-6.4	drug154	-6.3	drug541	-6.2
drug787	-6.4	drug160	-6.3	drug555	-6.2
drug854	-6.4	drug226	-6.3	drug633	-6.2
drug951	-6.4	drug240	-6.3	drug664	-6.2
drug993	-6.4	drug249	-6.3	drug683	-6.2
drug999	-6.4	drug348	-6.3	drug708	-6.2
drug1058	-6.4	drug402	-6.3	drug724	-6.2
drug1104	-6.4	drug426	-6.3	drug857	-6.2
drug1246	-6.4	drug431	-6.3	drug949	-6.2

drug1457	-6.4	drug433	-6.3	drug993	-6.2
drug6	-6.3	drug539	-6.3	drug999	-6.2
drug7	-6.3	drug727	-6.3	drug1319	-6.2
drug15	-6.3	drug783	-6.3	drug1400	-6.2
drug132	-6.3	drug841	-6.3	drug1407	-6.2
drug154	-6.3	drug859	-6.3	drug1494	-6.2
drug194	-6.3	drug1013	-6.3	drug14	-6.1
drug202	-6.3	drug1352	-6.3	drug38	-6.1
drug211	-6.3	drug1437	-6.3	drug51	-6.1
drug222	-6.3	drug1484	-6.3	drug153	-6.1
drug271	-6.3	drug34	-6.2	drug195	-6.1
drug311	-6.3	drug161	-6.2	drug221	-6.1
drug358	-6.3	drug163	-6.2	drug240	-6.1
drug372	-6.3	drug205	-6.2	drug255	-6.1
drug554	-6.3	drug254	-6.2	drug424	-6.1
drug595	-6.3	drug258	-6.2	drug515	-6.1
drug725	-6.3	drug273	-6.2	drug561	-6.1
drug842	-6.3	drug278	-6.2	drug595	-6.1
drug879	-6.3	drug412	-6.2	drug689	-6.1
drug925	-6.3	drug427	-6.2	drug700	-6.1
drug1009	-6.3	drug472	-6.2	drug718	-6.1
drug1013	-6.3	drug504	-6.2	drug797	-6.1
drug1034	-6.3	drug515	-6.2	drug802	-6.1
drug1068	-6.3	drug560	-6.2	drug803	-6.1
drug1157	-6.3	drug575	-6.2	drug859	-6.1
drug1201	-6.3	drug597	-6.2	drug1003	-6.1
drug1235	-6.3	drug772	-6.2	drug1090	-6.1
drug1341	-6.3	drug802	-6.2	drug1316	-6.1
drug1494	-6.3	drug857	-6.2	drug66	-6
drug37	-6.2	drug902	-6.2	drug67	-6
drug66	-6.2	drug903	-6.2	drug106	-6
drug195	-6.2	drug932	-6.2	drug108	-6
drug224	-6.2	drug959	-6.2	drug122	-6
drug249	-6.2	drug1054	-6.2	drug123	-6
drug255	-6.2	drug1080	-6.2	drug132	-6
drug390	-6.2	drug1089	-6.2	drug156	-6
drug396	-6.2	drug1157	-6.2	drug163	-6
drug432	-6.2	drug1234	-6.2	drug176	-6
drug515	-6.2	drug1264	-6.2	drug205	-6
drug590	-6.2	drug1290	-6.2	drug217	-6
drug670	-6.2	drug1314	-6.2	drug237	-6
drug683	-6.2	drug1375	-6.2	drug249	-6
drug742	-6.2	drug1423	-6.2	drug263	-6
drug772	-6.2	drug1443	-6.2	drug297	-6
drug802	-6.2	drug63	-6.1	drug377	-6
drug835	-6.2	drug241	-6.1	drug425	-6
drug1003	-6.2	drug287	-6.1	drug467	-6

drug1024	-6.2	drug354	-6.1	drug552	-6
drug1326	-6.2	drug491	-6.1	drug612	-6
drug122	-6.1	drug561	-6.1	drug613	-6
drug252	-6.1	drug889	-6.1	drug657	-6
drug337	-6.1	drug979	-6.1	drug686	-6
drug539	-6.1	drug1017	-6.1	drug725	-6
drug561	-6.1	drug1055	-6.1	drug902	-6
drug682	-6.1	drug1058	-6.1	drug923	-6
drug785	-6.1	drug1175	-6.1	drug925	-6
drug841	-6.1	drug1235	-6.1	drug1013	-6
drug857	-6.1	drug1353	-6.1	drug1306	-6
drug1017	-6.1	drug2	-6	drug1314	-6
drug1316	-6.1	drug167	-6	drug1443	-6
drug225	-6	drug196	-6	drug63	-5.9
drug226	-6	drug425	-6	drug116	-5.9
drug239	-6	drug432	-6	drug194	-5.9
drug254	-6	drug516	-6	drug203	-5.9
drug258	-6	drug672	-6	drug213	-5.9
drug377	-6	drug1158	-6	drug258	-5.9
drug413	-6	drug1260	-6	drug426	-5.9
drug528	-6	drug1419	-6	drug539	-5.9
drug555	-6	drug1494	-6	drug800	-5.9
drug596	-6	drug132	-5.9	drug828	-5.9
drug672	-6	drug176	-5.9	drug992	-5.9
drug686	-6	drug195	-5.9	drug1024	-5.8
drug700	-6	drug203	-5.9	drug1228	-5.8
drug903	-6	drug239	-5.9	drug1229	-5.8
drug966	-6	drug413	-5.9	drug16	-5.7
drug1319	-6	drug554	-5.9	drug37	-5.7
drug1432	-6	drug595	-5.9	drug161	-5.7
drug163	-5.9	drug670	-5.9	drug167	-5.7
drug253	-5.9	drug800	-5.9	drug253	-5.7
drug295	-5.9	drug816	-5.9	drug311	-5.7
drug349	-5.9	drug999	-5.9	drug357	-5.7
drug475	-5.9	drug1362	-5.9	drug396	-5.7
drug718	-5.9	drug16	-5.8	drug842	-5.7
drug789	-5.9	drug91	-5.8	drug2	-5.6
