# Supplementary material for: Repurposing Anti-Dengue Compounds against Monkeypox Virus Targeting Core Cysteine Protease
Source: Biomedicines. 2023 Jul 18;11(7):2025. doi: 10.3390/biomedicines11072025 (PMC10377189; doi:10.3390/biomedicines11072025)
Supplement: Supplementary file 1 [file biomedicines-11-02025-s001.zip › Supplemetary Figures S1-5.pdf]

(a)

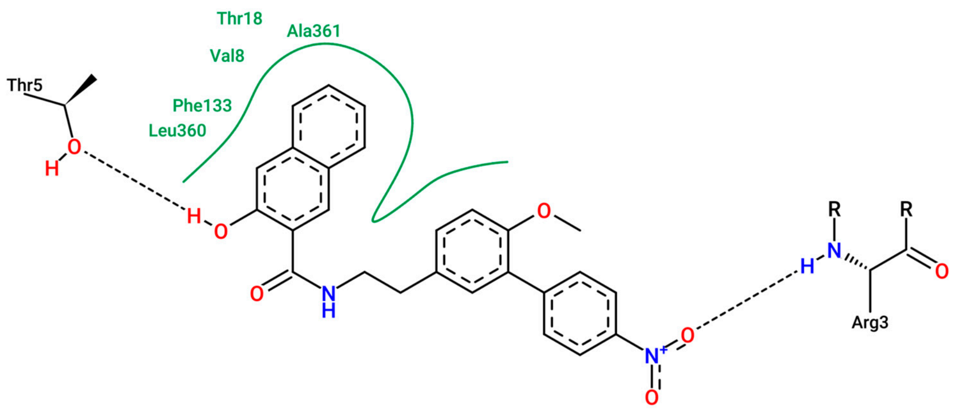

(b)

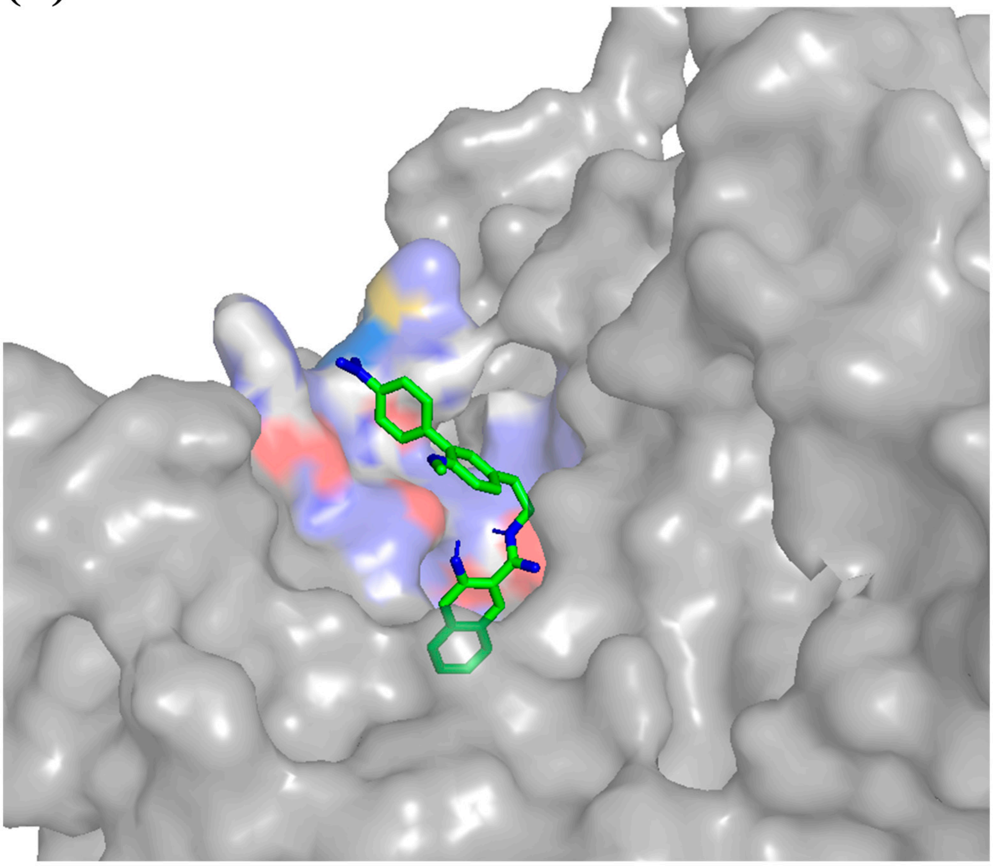

Supplementary Figure S1. The 2D and 3D representations of the binding interactions of the complexes (a, b) Cluster1 and TTP-6171 (control).

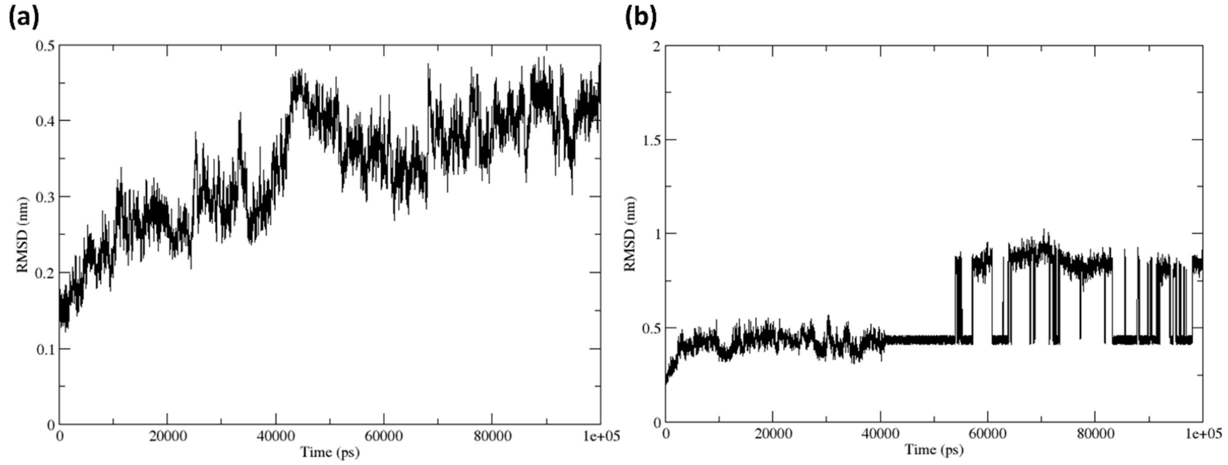

Supplementary Figure S2. The RMSD of the protein Cα-atoms and the ligand for the protein-ligand complex of Cluster1 and CHEMBL4549312.

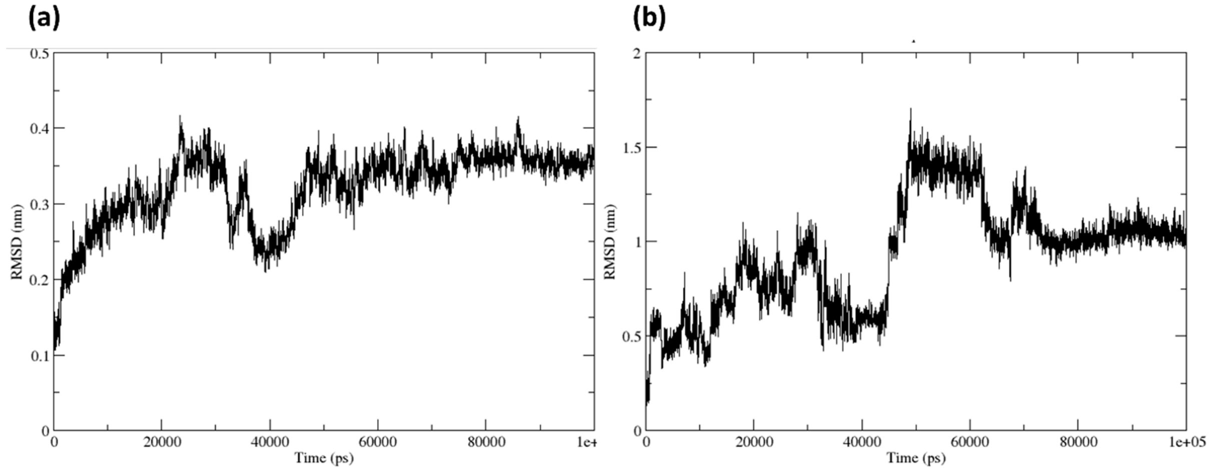

Supplementary Figure S3. The RMSD of the protein Cα-atoms and the ligand for the protein-ligand complex of Cluster2 and TTP-6171 (control).

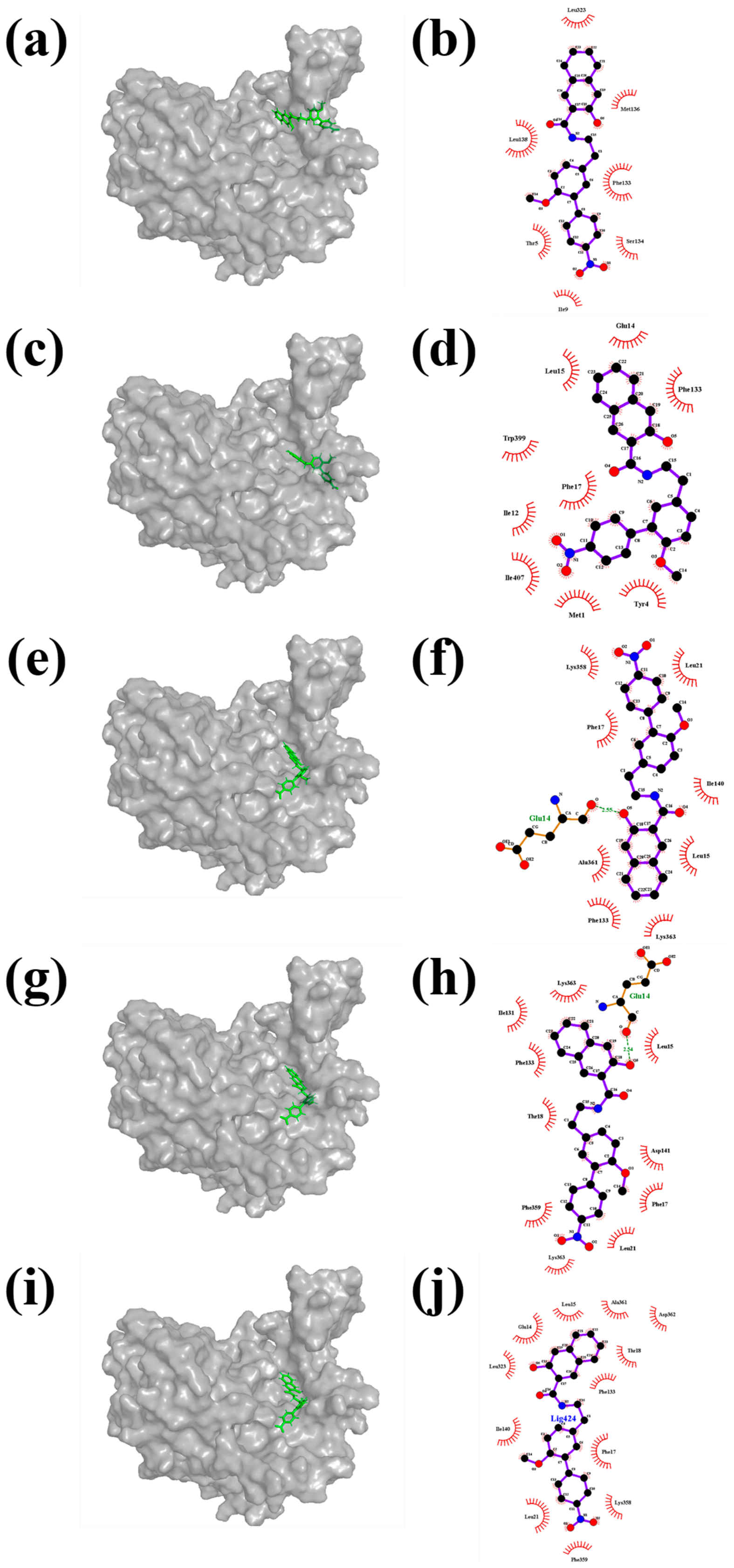

Supplementary Figure S4: The 3D and 2D representations of the binding interactions of the Cluster-2 and TTP-6171 (control) complex during the 100 ns MD simulation at the (a, b) 60 ns, (c, d) 70 ns, (e, f) 80 ns, (g, h) 90 ns and (i,j) 100 ns conformations.

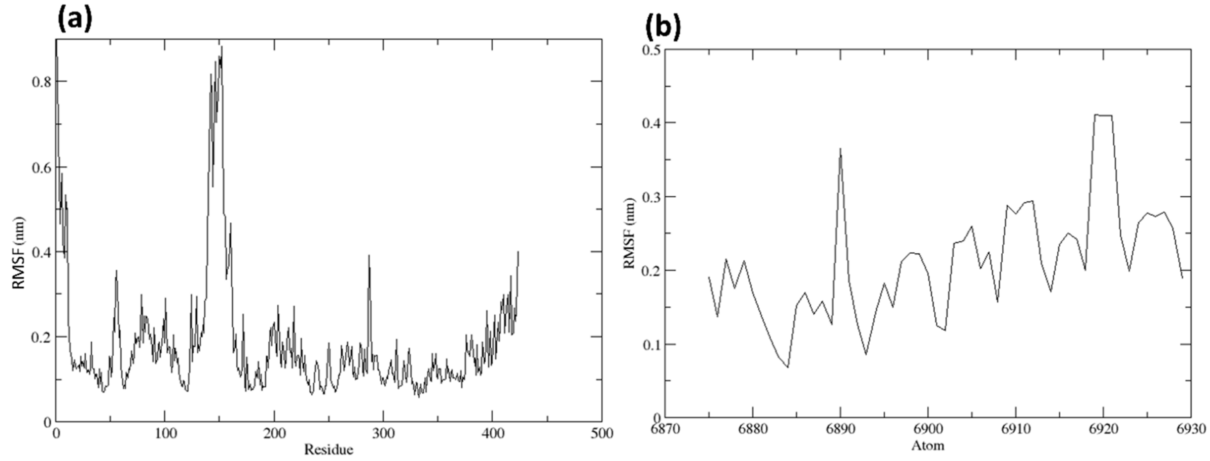

Supplementary Figure S5. The RMSF of the protein Cα-atoms and the ligand for the protein-ligand complex of Cluster2 and TTP-6171 (control).
